# Dominant suppressor genes of p53-induced apoptosis in *Drosophila melanogaster*

**DOI:** 10.1093/g3journal/jkae149

**Published:** 2024-07-10

**Authors:** Tamás Szlanka, Tamás Lukacsovich, Éva Bálint, Erika Virágh, Kornélia Szabó, Ildikó Hajdu, Enikő Molnár, Yu-Hsien Lin, Ágnes Zvara, Ildikó Kelemen-Valkony, Orsolya Méhi, István Török, Zoltán Hegedűs, Brigitta Kiss, Beáta Ramasz, Laura M Magdalena, László Puskás, Bernard M Mechler, Adrien Fónagy, Zoltán Asztalos, Gábor Steinbach, Michal Žurovec, Imre Boros, István Kiss

**Affiliations:** Institute of Biochemistry, HUN-REN Biological Research Centre, 6726 Szeged, Hungary; Biology Centre, Czech Academy of Sciences, 37005 České Budějovice, Czech Republic; Institute of Genetics, HUN-REN Biological Research Centre, 6726 Szeged, Hungary; Brain Research Institute, University of Zurich, 8057 Zurich, Switzerland; Institute of Biochemistry, HUN-REN Biological Research Centre, 6726 Szeged, Hungary; Institute of Genetics, HUN-REN Biological Research Centre, 6726 Szeged, Hungary; Institute of Biochemistry, HUN-REN Biological Research Centre, 6726 Szeged, Hungary; Biology Centre, Czech Academy of Sciences, 37005 České Budějovice, Czech Republic; Institute of Genetics, HUN-REN Biological Research Centre, 6726 Szeged, Hungary; Institute of Genetics, HUN-REN Biological Research Centre, 6726 Szeged, Hungary; Department of Developmental Genetics, German Cancer Research Centre, 69120 Heidelberg, Germany; Institute of Genetics, HUN-REN Biological Research Centre, 6726 Szeged, Hungary; Institute of Genetics, HUN-REN Biological Research Centre, 6726 Szeged, Hungary; Biology Centre, Czech Academy of Sciences, 37005 České Budějovice, Czech Republic; Laboratory of Functional Genomics, Core Facility, HUN-REN Biological Research Centre, 6726 Szeged, Hungary; Cellular Imaging Laboratory, Core Facility, HUN-REN Biological Research Centre, 6726 Szeged, Hungary; Institute of Genetics, HUN-REN Biological Research Centre, 6726 Szeged, Hungary; Department of Developmental Genetics, German Cancer Research Centre, 69120 Heidelberg, Germany; Bioinformatics Laboratory, Core Facility, HUN-REN Biological Research Centre, 6726 Szeged, Hungary; Department of Biochemistry and Medical Chemistry, Medical School, University of Pécs, 7624 Pécs, Hungary; Institute of Genetics, HUN-REN Biological Research Centre, 6726 Szeged, Hungary; Institute of Genetics, HUN-REN Biological Research Centre, 6726 Szeged, Hungary; Institute of Genetics, HUN-REN Biological Research Centre, 6726 Szeged, Hungary; Laboratory of Functional Genomics, Core Facility, HUN-REN Biological Research Centre, 6726 Szeged, Hungary; Department of Developmental Genetics, German Cancer Research Centre, 69120 Heidelberg, Germany; Centre for Agricultural Sciences, Plant Protection Institute, 1022 Budapest, Hungary; Institute of Biochemistry, HUN-REN Biological Research Centre, 6726 Szeged, Hungary; Aktogen Hungary Ltd., 6726 Szeged, Hungary; Cellular Imaging Laboratory, Core Facility, HUN-REN Biological Research Centre, 6726 Szeged, Hungary; Biology Centre, Czech Academy of Sciences, 37005 České Budějovice, Czech Republic; Institute of Biochemistry, HUN-REN Biological Research Centre, 6726 Szeged, Hungary; Institute of Genetics, HUN-REN Biological Research Centre, 6726 Szeged, Hungary

**Keywords:** apoptosis, p53, suppression, activating insertional mutagenesis, *Drosophila*

## Abstract

One of the major functions of programmed cell death (apoptosis) is the removal of cells that suffered oncogenic mutations, thereby preventing cancerous transformation. By making use of a Double-Headed-EP (*DEP*) transposon, a *P* element derivative made in our laboratory, we made an insertional mutagenesis screen in *Drosophila melanogaster* to identify genes that, when overexpressed, suppress the *p53*-activated apoptosis. The *DEP* element has Gal4-activatable, outward-directed *UAS* promoters at both ends, which can be deleted separately in vivo. In the *DEP* insertion mutants, we used the *GMR-Gal4* driver to induce transcription from both *UAS* promoters and tested the suppression effect on the apoptotic rough eye phenotype generated by an activated *UAS-p53* transgene. By *DEP* insertions, 7 genes were identified, which suppressed the *p53*-induced apoptosis. In 4 mutants, the suppression effect resulted from single genes activated by 1 *UAS* promoter (*Pka-R2*, *Rga*, *crol*, and *Spt5*). In the other 3 (*Orct2*, *Polr2M*, and *stg*), deleting either *UAS* promoter eliminated the suppression effect. In qPCR experiments, we found that the genes in the vicinity of the *DEP* insertion also showed an elevated expression level. This suggested an additive effect of the nearby genes on suppressing apoptosis. In the eukaryotic genomes, there are coexpressed gene clusters. Three of the *DEP* insertion mutants are included, and 2 are in close vicinity of separate coexpressed gene clusters. This raises the possibility that the activity of some of the genes in these clusters may help the suppression of the apoptotic cell death.

## Introduction

Cells seriously damaged by stress or not needed in development are removed by the process of programmed cell death, a genetically regulated “suicide” of cells (apoptosis, pyroptosis, ferroptosis, necroptosis, and entosis; Aubrey *et al.* 2018b; Liang *et al.* 2021; Yan *et al.* 2021; Yu *et al.* 2021; Bertheloot *et al.* 2021; Rizzotto *et al.* 2021). In the process of apoptosis (Pakos-Zebrucka *et al.* 2016; Voss and Strasser 2020), the transcription factor p53 is the central mediator that directly or indirectly controls the expression of an estimated 3,000 genes (Sammons *et al.* 2020). With its several isoforms, it is involved in the maintenance of cellular homeostasis, coordinating cell survival and senescence, stem cell renewal and differentiation, programmed cell death, etc. (Anbarasan and Bourdon 2019; Mehta *et al.* 2021).

A major activator of the *p53* gene is the genetic stress (DNA damage, oncogenic mutations, and aneuploidy), which can lead to uncontrolled cell proliferation and cancer. In more than 50% of tumors, the *p53* gene has missense mutations, mostly at 6 “hot spot” amino acid residues located in the DNA-binding domain. Some of these specific single amino acid substitutions are classified as gain-of-function (GOF) mutations that drive tumorigenesis (Alvarado-Ortiz *et al.* 2020); however, other studies reported that most of them act as dominant-negative effect or loss-of-function mutations (Aubrey *et al.* 2018a; Boettcher *et al.* 2019; Wang *et al.* 2024).

With respect to the cancerous transformation, the negative regulators/suppressors of *p53* and/or apoptosis are of particular importance. Such genes, like members of the BCL-2 family (Singh *et al.* 2019; Kaloni *et al.* 2023), the IAP family (Cetraro *et al.* 2022), MDM2 (Hou *et al.* 2019), API5 (Abbas *et al.* 2024), and DDIAS (Im *et al.* 2023), under normal conditions, prevent unwanted cell death, and they play important roles in maintaining the cellular and organismal homeostasis. However, their abnormally elevated expression may interfere with the normal regulation of p53 and apoptosis, opening the gate to abnormal cell proliferation and cancer progression (Peng *et al.* 2022).

The discovery of a *p53 Drosophila* orthologous gene *Dmp53* (Brodsky *et al.* 2000; Ollmann *et al.* 2000) revealed that the overall amino acid sequence homology of Dmp53 with the mammalian Tp53 is not particularly high. However, their protein structure, DNA-binding domain sequence, function, and even interaction network are highly similar and evolutionarily well conserved; therefore, the results gained in *Drosophila* can easily be interpreted for the mammalian system (D’Brot *et al.* 2017; Zhou 2019; Chakravarti *et al.* 2022). Specific functions for isoforms Dmp53A and Dmp53B are also reported in somatic, germline, and polyploid tissues of *Drosophila* (Zhang *et al.* 2014, 2015; Chakravarti *et al.* 2022).

The strategy of selectively activating random genes by the insertion of *P* element constructs that carry Gal4-inducible promoters, e.g. the *EP* element (Rørth 1996) or the *GS* construct (Toba *et al.* 1999), was successfully applied previously for the analysis of complex biological functions in the fruit fly. To recover dominant suppressors of *p53*-induced apoptosis, we made a GOF screen by making use of the *DEP* element, which is similar to *EP* but significantly improved, made in our laboratory. We identified 7 insertion mutants that, when overexpressed, significantly suppressed the apoptotic effect in the eyes, i.e. the rough eye (*r.e.*) phenotype, in the *GMR-Gal4>DEP*, *UAS-p53* combination. In 3 of them, however, the activation of 1 gene was not enough to exert the suppression effect. As the genes around the *DEP* insertion are also activated to some extent, they might also contribute to the suppression of *r.e.*

## Materials and methods

### Fly cultures and stocks

Fly cultures were kept on standard cornmeal–yeast–agar medium at 25°C if not otherwise stated. The genetic combinations tested were established by standard genetic crosses on *w* homozygous background.

The following stocks were received from the BDSC Stock Center, Bloomington, Indiana:

- ***P(Δ2-3)***: *ry[506] P{ry[+t7.2]=Delta2-3}99B*
- **GMR-Gal4**: w[*]; P{w[+mC]=GAL4-ninaE.GMR}12
- ***UAS-p53***: *y[1] w[1118]*; *P{w[+mC]=UAS-p53.Ex}3* (expresses the A isoform of *p53*)
- ***yw; MKRS, FLP/TM6B, Cre***: *y[1] w[67c23]; MKRS, P{ry[+t7.2]=hsFLP}86E/TM6B, P{w[+mC]=Crew}DH2, Tb[1*]
- ***UAS-stg***: *w[1118]; P{w[+mC]=UAS-stg.N}16/CyO, P{ry[+t7.2]=sevRas1.V12}FK1*
- w[*]; T(2;3)ap[Xa], ap[Xa]/CyO; TM6

The shortened genotypes in bold preceding the complete ones represent the name used in the text. *UAS-Spt5* was a kind gift from Ruth Palmer. Transgenic *RNAi* stocks were received from the NIG-FLY (Mishima), VDRC (Vienna; Dietzl *et al.* 2007), and BDSC (Bloomington) collections. The *w, DEP* homozygous stock used for the transposon mutagenesis was created in our laboratory (see below). In the description of the genetic constructs, we followed the terms of the last updates of FlyBase (Öztürk-Çolak *et al.* 2024)

### Construction of the *DEP* activating transposon

**Fig. 1.**
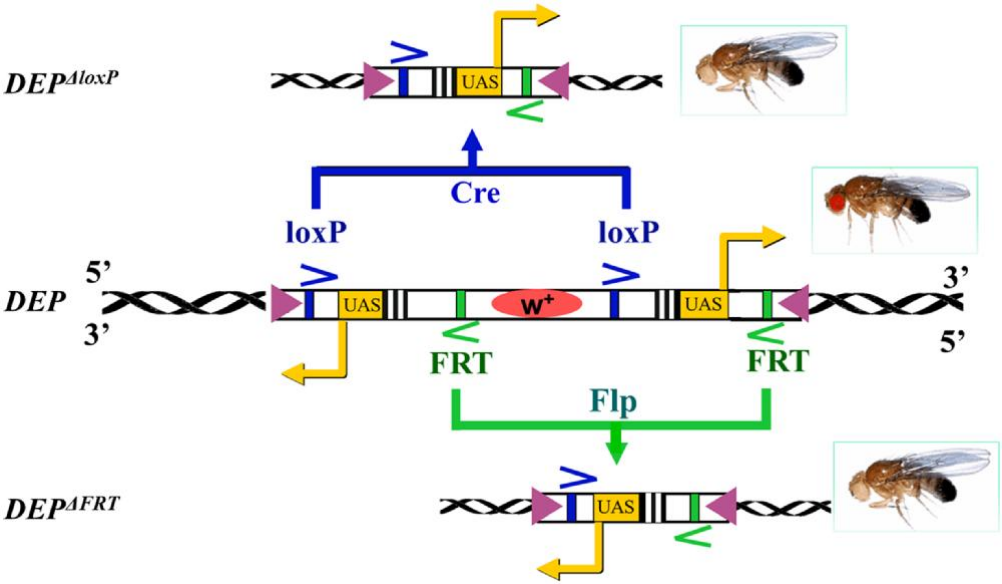
Structure of the *DEP* element and selective deletion of the *UAS* promoters. The 2 outward-directed *UAS* promoters are located at the ends of the *mini-w^+^ DEP* construct. The *UAS* promoters located at the 5′- and the 3′-ends are flanked by a pair of *FRT* and *loxP* sites, respectively. Each one of the *UAS* promoters together with the *mini-w^+^* marker can selectively be deleted in vivo by the Cre and Flp recombinases (leaving the other *UAS* promoter intact) resulting Δ*loxP* and *ΔFRT* derivatives, respectively. The rectangular arrows at the *UAS* sites show the directions of the Gal4-induced transcription from the *UAS* promoters, and the triangles at the ends of the *DEP* construct represent the terminal repeats of the *DEP* element.

The *pDEP* construct was made in our laboratory as follows: at first, we replaced the entire gene trap cassette in the backbone of *pGT1* vector (Lukacsovich *et al.* 2001) with the *mini-white^+^* (*m-w^+^*) gene of *pCasper2*. This step resulted in unique NotI as well as XhoI restriction sites next to the 5′ and 3′ *P* element ends, respectively. Using these sites, 2 multicloning sites (MCSs) containing several unique restriction sites were inserted in both sides of the *m-w^+^* gene by ligating synthetic double-stranded oligonucleotides into the locations. The *5xUAS-hsp70-*core promoter fragment — from the *pUAST* vector (Brand and Perrimon 1993) — and the *loxP* and *FRT* sequences were then inserted in the desired orientations into the MCSs to get the final *DEP* construct (Fig. 1). A detailed description of the steps of construction is available upon request. As Fig. 1 shows, the sequence unit containing the *UAS* promoter at the 5′ end of *DEP* and the *m-w^+^* gene together are flanked by *FRT* sequences (*UAS^FRT^*) while the 3′ *UAS* and the *m-w^+^* are between 2 *loxP* sites (*UAS^loxP^*). This arrangement makes the *UAS* promoters selectively deletable in vivo by the FLP or Cre recombinases. The *pDEP* construct was microinjected along with the *Δ2-3* transposase helper plasmid into *w^1118^* syncytial blastoderm-stage embryos by using standard techniques. Surviving adults were crossed again to *w^1118^* homozygous flies, and in the next generation, transformants were screened for their red eye color, and X chromosomal insertions were selected.

### Genetic screen for dominant modifiers of the *p53*-induced apoptosis

As shown in Supplementary Fig. 1, female flies carrying the *DEP* element on the X chromosome were crossed to males of the *P(Δ2-3)* jumpstarter stock producing the *P* element transposase (Robertson *et al.* 1988). Remobilized by the transposase, the *DEP* element “jumps out” of the X chromosome and gets inserted at new sites in the genome. Males carrying the new insertions in their germline were crossed to females of a *T(2;3)* translocation balancer *w/w; T(2;3)ap[Xa], ap[Xa]/CyO; TM6*. In the next generation, male offspring (*w/Y*) have white eyes, except those which carry new autosomal *DEP* insertions and have colored eyes by the *m-w^+^* expression. Single males with colored eyes were simultaneously crossed to *T(2;3)* translocation balancer females (see above) and homozygous “tester” females of *w; GMR-Gal4; UAS-p53* genotype. To select against the *P(Δ2-3)* transposase source, we used those males only, which lacked any sign of eye color mosaicism. In the next generation, if the *DEP* insertion mutant activated by the *GMR-Gal4* driver suppressed the *p53*-induced *r.e.* phenotype, red-eyed males carrying the new *DEP* suppressor mutation above *CyO* or *TM6* balancer were crossed again to the appropriate balancer females. Through serial crosses to balancer stocks, the new insertions on the second or third chromosomes were isolated as homozygous mutant lines.

### Determination of *DEP* insertion sites

Inverse PCR was performed according to the protocol described previously (Kyriacou 2000), with some modification. Shortly, genomic DNA of approximately 10 flies carrying a *DEP* insertion was extracted and digested with the restriction enzyme HpaII (NEB), and after phenol–chloroform extraction, the resulting fragments were ligated with T4 ligase (NEB) for 2 h at room temperature to circularize them. Two microliters out of the 20 μL ligation mixture was used as template in the PCR reaction. The PCR reactions were performed using Taq DNA Polymerase (QIAGEN) with the Taq PCR buffer, 1.5 mM MgCl_2_, 0.2 mM dNTPs, and a primer pair specific to the 3′P-end of the *DEP* element: P3′Fw1 that hybridizes between nucleotide positions 106 and 131 in the *DEP* vector (GTCTGAGTGAGACAGCGATATGATTG) and P3′Rev1 that binds to the vector between positions 75 and 51 (CACTCGCACTTATTGCAAGCATACG) on the complementary strand, both at 0.5 μM final concentration. The sample was cycled 35 times for 30 s at 95°C, 30 s at 58°C, and 1 min at 72°C. One microliter of the resulted reaction mixture was used as template for a second round of PCR reaction using the following nested primer pair: P3′Fw2 that hybridizes between nucleotide positions 131 and 154 (GTTGATTAACCCTTAGCATGTCCG) and P3′Rev2 that binds to the vector between positions 50 and 28 (TTAAGTGGATGTCTCTTGCCGAC) on the complementary strand, again at 0.5 μM final concentration. The second round reaction was performed under the same conditions as the first round except the annealing temperature was elevated to 60°C.

After purification (QIAquick, QIAGEN), the PCR product was sequenced with primers P3′Fw2 and/or P3′Rev2. Sequence data were blasted to FlyBase (FB2024_02, released 2024 April 23) to identify the genomic region carrying *DEP* insertion. Insertion points were verified in a third round of PCR reaction using a primer specific to the 5′P-end of the *DEP* element (P5′Fw) that hybridizes between nucleotide positions 5483 and 5504 in the *DEP* vector (GTATACTTCGGTAAGCTTCGGC) and a primer specific to each of the relevant genomic regions identified. The *DEP* insertion site sequences are given in Supplementary Table 1.

### Confocal microscopy

Imaginal eye-antennal disks complexed to CNS from the third instar larvae were dissected and mounted in PBS, and native fluorescent signal of GFP was detected by Leica SP5 AOBS confocal laser scanning microscope (Leica, Germany). The images of compared eye disks of the *DEP*-bearing genotypes and their corresponding controls were captured from the same slide and at the same time within 1 h. We used a 488-nm argon laser for the excitation of the fluorescent signal of GFP, and the emission signals were detected by spectral detector in 500-590 nm range. The optical sections of the samples for quantitative analysis were taken using HCX PL FLUOTAR 5×/0.15 objective; image size: 1,024 pixel × 1,024 pixel, 3,100 μm × 3,100 μm, and pinhole 70 μm. Some selected samples were acquired for detailed images using HCX PL FLUOTAR 40×/0.75 objective; image size: 1,024 pixel × 1,024 pixel, 388 μm × 388 μm, line average 3, and pinhole 113 μm. The images were analysed by the FIJI software (Schindelin *et al.* 2012). The fluorescence intensities of the Z-sections were averaged using Z-projection, and mean/std was calculated from the pixel values higher than 25 for every kind of sample. (Background pixels less intensive than 25 were marked as NaN [not a number] and excluded from the calculation.)

### Selective in vivo deletion of the *UAS* promoters in the *DEP* insertion mutants

To induce promoter deletion in the *DEP* element, the suppressor mutants (*suppr^DEP^*) were crossed to *yw; MKRS, FLP/TM6B, Cre* flies, where the *MKRS* and *TM6B* balancer chromosomes carry heat-inducible transgenes of the FLP and Cre site-specific recombinases, respectively. The recombinases were induced by heat shock (37°C, 2 h) in the second instar larvae of the F_1_ generation. The male F_1_ flies carrying the *MKRS* or *TM6B* chromosomes were separately crossed to homozygous *w* balancer stocks. Because the *UAS* (along with the coupled promoter) and the *mini-w^+^* marker were removed together, the *UAS*-deleted flies (*suppr^DEPΔFRT^* or *suppr^DEPΔloxP^*) in the next generation could be recognized by the white eye color (Fig. 1).

### Silencing the suppressor genes with *RNAi*

To test whether silencing the *DEP*-bearing gene really weakened the suppression of apoptosis, we constructed *Drosophila* stocks carrying an *RNAi* transgene and the corresponding *DEP* suppressor mutant on separate autosomes. These stocks were crossed to the *w; GMR-Gal4; UAS-p53* homozygous “tester” stock. Among the F_1_ offspring, we evaluated the *r.e.* phenotype of the flies that carried the *DEP* suppressor mutant together with the specific *RNAi* silencing construct and the *UAS-p53* transgene, all of them driven by the *GMR-Gal4* driver: *GMR-Gal4>suppr^DEP^*, *UAS-p53*, *UAS-RNAi*.

### RNA preparation and RT-qPCR

Total RNA from 20 heads of 3-day-old *Drosophila* adults for each genetic combination was purified using the RNA isolation kit of Macherey-Nagel (Macherey-Nagel, Düren, Germany) according to the manufacturer's instructions. One microgram of total RNA was reverse transcribed using the High-Capacity cDNA Archive Kit (Thermo Fisher Scientific, Waltham, MA, USA) according to the manufacturer's instructions in 20 μL final volume at 37°C for 2 h following a preincubation at room temperature for 10 min. After inactivating the enzyme at 75°C for 10 min, the reaction mixture was diluted 30 times. One microliter of the diluted reaction mix was used as template in the qPCR.

The reaction was performed with gene-specific primers and HOT FIREPol EvaGreen qPCR Mix Plus (ROX) (Solis BioDyne) according to the manufacturer's instructions at a final primer concentration of 250 nM in Eco Real-Time PCR System (Illumina) under the following conditions: 15 min at 95°C, 40 cycles of 95°C for 15 s, 60°C for 20 s, and 72°C for 20 s. Parts of the reactions were performed using 2× qPCRBIO SyGreen Mix Lo-ROX (PCR Biosystems) according to the manufacturer's instructions at a final primer concentration of 250 nM in RotorGene RG3000 (Corbett Research) qPCR system under the following conditions: 2 min at 95°C, 35 cycles of 95°C for 5 s, and 60°C for 30 s. Melt curve analysis was done after each reaction to check the quality of the products. Primers were designed online using the Roche Universal Probe Library Assay Design Center or the Integrated DNA Technologies qPCR Assay Design RealTime PCR Tool. The primers used to detect *p53* mRNA were reported earlier (Pardi *et al.* 2011). Individual threshold cycle (Ct) values were normalized to Ct values of *fzr* and *FoxK* internal control genes. Relative gene expression levels between induced and control genotypes are presented as fold change values calculated using the formula (fold change = 2^ΔΔCt^), according to the ΔΔCt method (Livak and Schmittgen 2001). For comparation of induced *p53* mRNA levels between *GMR-Gal4>suppr^DEP^*, *UAS-p53* genotypes and *GMR-Gal4>UAS-p53* ΔΔCt values are directly presented. Primers used in qPCR analysis are listed in Supplementary Table 2.

### Statistical analysis of RT-qPCR data

RNA samples were prepared and tested in 3 biological replicates (*n* = 3) for each genetic combination. Statistical comparison of normalized Ct (ΔCt) values of control and induced genotypes was done by Student's *t*-test (2-tailed, unequal variance). Results are summarized in Supplementary Table 3.

## Results

### Isolation and characterization of the mutants carrying the *DEP* insertions

Overexpression of *Dmp53* in the whole body is lethal. To isolate dominant suppressor mutants of the *p53*-induced apoptosis, we took advantage of the *GMR-Gal4* driver, which expresses the Gal4 mainly in the eye (Freeman 1996; Neufeld *et al.* 1998; Ray and Lakhotia 2015). In heterozygous *GMR-Gal4>UAS-p53* flies (*GMR-Gal4/+; UAS-p53/+*), the elevated expression of *p53A* isoform causes extensive apoptotic cell death in the eye imaginal disks and results in smaller than normal adult eyes with highly disorganized ommatidial arrays: “*r.e.*” phenotype (Ollmann *et al.* 2000; Jin *et al.* 2000; Kim *et al.* 2011), as also shown in Fig. 3 (compare a and b). It has to be noted that in the *GMR-Gal4/+* heterozygous condition, the *GMR-Gal4* driver alone does not show any *r.e.* phenotype (Fig. 3a′). As the flies showing the *r.e.* phenotype are viable and fertile (Kramer and Staveley 2003), we built our activating mutagenesis screen on this approach (for the details, see Supplementary Fig. 1). For the mutagenesis, we used the *DEP P* element construct with 2 outward-directed *UAS*-coupled promoters (“*UAS* promoters”), 1 at each end (Fig. 1). As the Gal4 activates both *UAS* promoters, the transcription simultaneously starts in both directions from the insertion site.

**Fig. 2.**
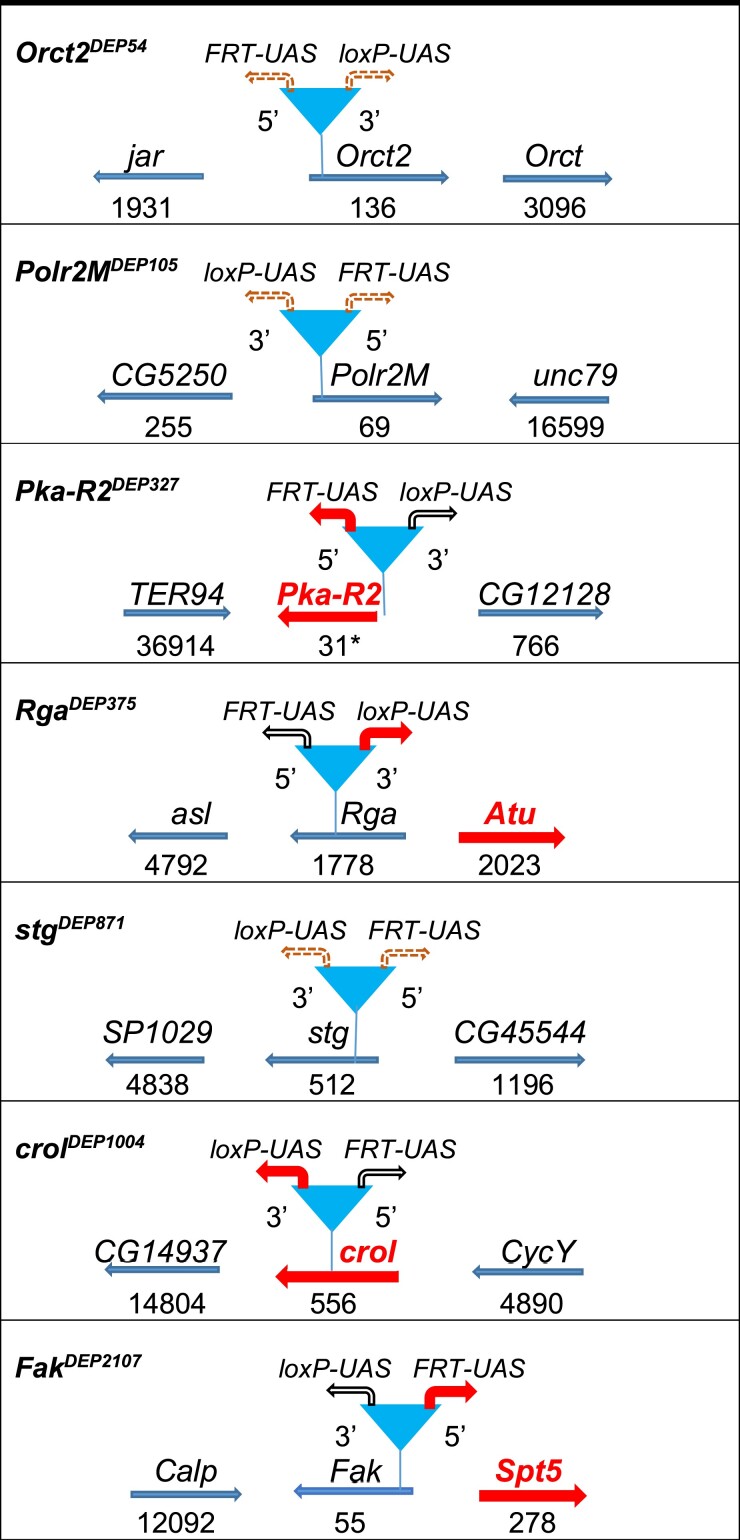
General features of *DEP* insertions and their genomic neighborhood. For the DNA sequence of the insertion site, see Supplementary Table 1. Arrows label the direction of gene transcription. Numbers below the arrows indicate the distance of the *DEP* insertion site in base pairs downstream from the gene's transcription start site. Asterisk denotes the distance is upstream from the gene's transcription start site. Thick arrows represent genes that are responsible for the suppression effect. The triangles represent the position of the *DEP* insertions. 5′*FRT-UAS* and 3′*loxP-UA*S with thick rectangular arrows mean the Gal4-activatable *UAS* promoter identified as the activator of the suppression of apoptosis. Dashed rectangular arrows mean that the apoptosis suppressor effect of neither *UAS* promoter can be determined unequivocally.

**Fig. 3.**
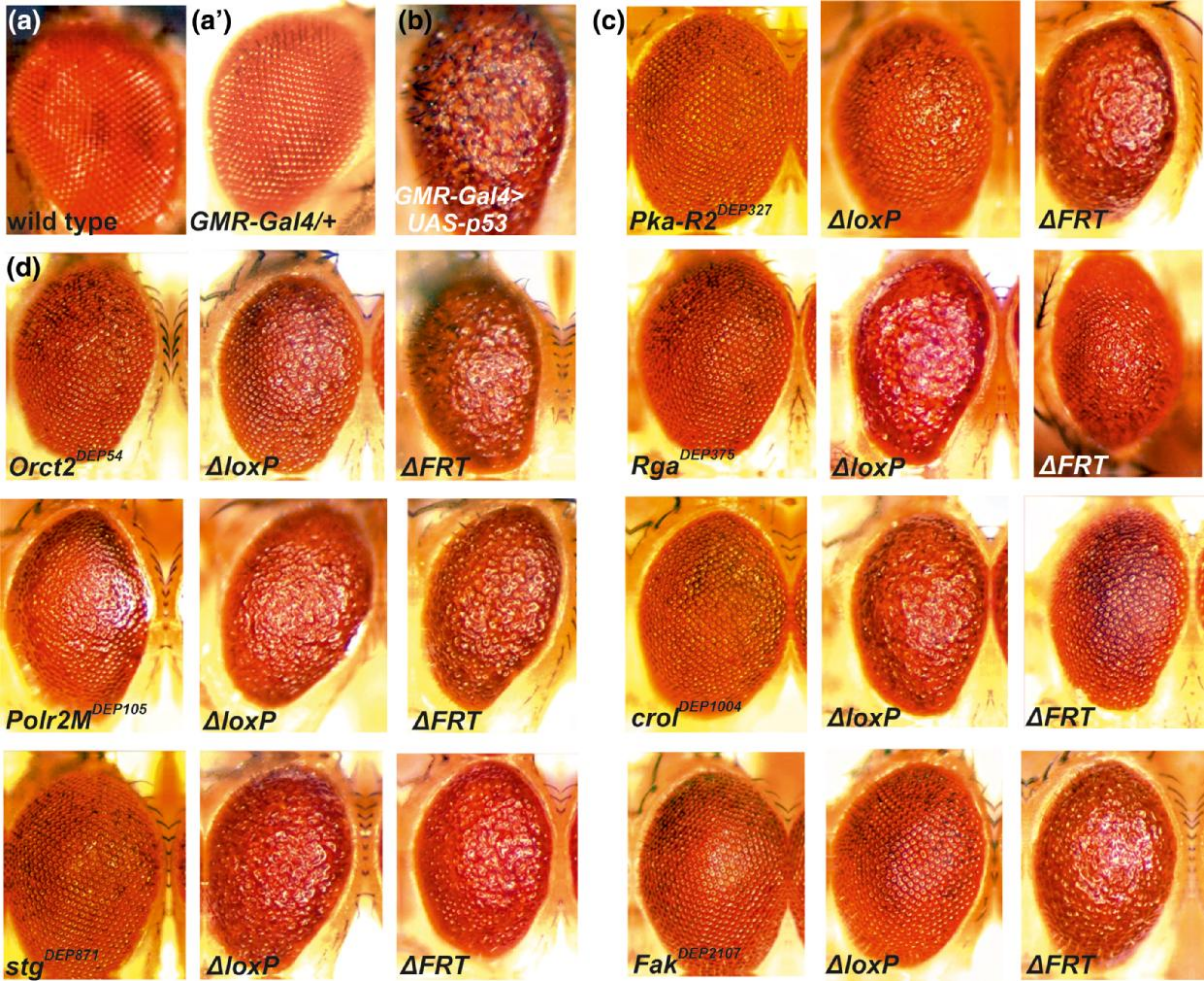
Effect of *Gal4*-activated suppressor gene mutants and their *UAS*-deleted derivatives on the *p53*-induced apoptotic *r.e.* phenotype. a) Wild-type adult eye. a′) Normal eye of *GMR-Gal4/+* heterozygote. b) *r.e.* phenotype of the *GMR-Gal4>UAS-p53*. c and d) Apoptosis suppression effect of *GMR-Gal4>suppr^DEP^, UAS-p53* combinations. Δ*loxP* and Δ*FRT* stand for the *UAS*-deleted *DEP* mutant derivatives *DEP^ΔloxP^* and *DEP^ΔFRT^*, respectively (see Fig. 1). c) Suppression of *r.e.* phenotype is caused by 1 of the 2 *UAS*s. d) Deletion of either one or the other *UAS* promoter results in the same *r.e.* phenotype, i.e. the apoptosis suppression effect cannot be definitely related to either *UAS* promoter.

Since the *P* element preferentially inserts near the 5′ end of the gene (Shilova *et al.* 2006), we expected that the induced downstream transcription from most *DEP* insertions would have resulted in enhanced gene expression. We searched for gene mutants (*suppr^DEP^*) that could suppress the *p53* overexpression-induced *r.e.* phenotype when activated by Gal4 in the genetic combination *GMR-Gal4>suppr^DEP^*, *UAS-p53*. Out of more than 2,000 insertions on the second and third chromosomes, we recovered 7 such mutants (Figs. 2 and 3). All of them showed strong suppressor effect producing weaker than grade 1 *r.e.* phenotype according to our arbitrary *r.e.* scale (Supplementary Fig. 2). By sequencing the DNA flanking the insertions in the mutant lines, we identified 7 genes (*Orct2*, *Polr2M*, *Pka-R2*, *Rga*, *stg/CDC25*, *crol*, and *Fak*) with the *DEP* transposon inserted near their 5′-end and also determined the orientation of the *DEP* elements (Fig. 2): in 3 out of 7, the *DEP* insertions are in the first exon (*Polr2M^DEP105^*, *stg^DEP871^*, and *Fak^DEP2107^*). In *Orct2^DEP54^*, the *DEP* insert is 129 bp downstream from the transcription start site in the unsplit gene. *Rga^DEP375^* has the insert in the first intron while *crol^DEP1004^* has it in the second exon. In *Pka-R2^DEP327^*, the *DEP* transposon is inserted upstream but near the 5′-end of the gene. As the *DEP* insertion sites are upstream relative to the translation start sites in all but 1 (*Polr2M^DEP105^*, 27 bp downstream from the translation start site) of the mutants, we supposed at first that the suppressor effect was a result of the Gal4-induced downstream transcription and overexpression of the gene. However, the transcription starting from a *UAS* promoter could also spread over the nearby genes. This assumption was tested by measuring the expression level of the neighbor genes by quantitative PCR and the selective deletion of the *UAS* promoters of the *DEP* element (see below).

It has to be noted that in all of the different genetic combinations tested, we used every component in heterozygous condition. The *GMR-Gal4>UAS-p53* flies showed a strong, characteristic *r.e.* phenotype that, at the same time, was sensitive enough to be readily modified by the Gal4-activated *DEP* suppressor mutants in the *GMR-Gal4>suppr^DEP^*, *UAS-p53* heterozygous flies (*w^1118^; GMR-Gal4/+; suppr^DEP^*/*UAS-p53*). These heterozygous combinations were able to detect even the weak combined effect of the genes near the *DEP* insertion (Fig. 3). To exclude the possibility that the insertions suppress the *r.e.* phenotype by simply reducing the ability of the *GMR-Gal4* driver to activate *UAS-p53*, we measured the *p53* mRNA level in *GMR-Gal4>suppr^DEP^*, *UAS-p53* flies by quantitative PCR and compared the results to that derived from the original *p53* overexpressing *GMR-Gal4>UAS-p53* flies. As Supplementary Fig. 3 shows, no substantial difference could be detected between the ΔΔCt values of the *GMR-Gal4>suppr^DEP^*, *UAS-p53* genotypes and that of the *GMR-Gal4>UAS-p53*. The small differences that are still detectable, however, do not correlate with the differences in the strength of suppression shown in Fig. 3. Furthermore, in 3 particular cases (*Orct2^DEP54^*, *Polr2M^DEP105^*, and *stg^DEP871^*), where the gene responsible for the suppressor effect could not be identified unequivocally (see below), in a “counter-screen,” we tested the effect of the insertions on the efficiency of *GMR-Gal4* to drive *UAS-GFP* in the eye-antennal disk of the third instar larvae. Supplementary Fig. 4 shows that there is no difference of substance between representative confocal images of *GMR-Gal4>suppr^DEP^*, *UAS-GFP* and *GMR-Gal4>UAS-GFP*. The quantitative analysis of the confocal images represented by bar chart in Supplementary Fig. 5 shows that strength of the GFP signal in the eye disks from *GMR-Gal4>suppr^DEP^*, *UAS-GFP* larvae does not differ substantially from that derived from *GMR-Gal4>UAS-GFP* larvae.

Altogether, these experiments prove that the suppressing effect of the *DEP* insertions on *r.e.* phenotype does not originate from their ability to weaken the strength of *GMR-Gal4* activation on *UAS-p53*.

### Determination of the genes responsible for apoptosis suppression

The *DEP* construct carries 2 outward-directed *UAS* promoters (Fig. 1), and the Gal4 simultaneously activates transcription from both. As a first assumption, one would expect that the *UAS* promoter, which initiates downstream transcription of the *DEP*-bearing gene, is responsible for the suppressor effect. To test this, the promoters were in vivo deleted separately by the FLP or Cre recombinases (see *Materials and methods*), and the mutant bearing the truncated *DEP* element (*DEP^ΔFRT^* or *DEP^ΔloxP^*) was crossed to homozygous *GMR-Gal4; UAS-p53* tester flies to see if the apoptosis suppression effect was lost or retained. As the results show, the mutants can be distributed into 2 groups. In the first one (*Pka-R2^DEP327^*, *Rga^DEP375^*, *crol^DEP1004^*, and *Fak^DEP2017^*), if deleting one *UAS* promoter abolishes the suppressor activity, then deleting the other one has weak or no effect (Figs. 2 and 3c). In *Pka-R2^DEP327^* and *crol^DEP1004^*, the downstream transcription of the gene responsible for the apoptosis suppression is initiated by the *FRT*-deletable *UAS^FRT^* and the Cre-deletable *UAS^loxP^* promoter, respectively. In the case of *Rga^DEP375^* and *Fak^DEP2017^*, the orientation of the *UAS* responsible for the suppression effect points to the upstream direction from the *DEP* insertion, toward the neighbor genes *Atu* and *spt5*, respectively (Fig. 2). In accordance with this, *Gal4*-induced expression of a *UAS-Fak* transgene remained ineffective (not shown).

In the mutants of the second group, *Orct2^DEP54^*, *Polr2M^DEP105^*, and *stg^DEP871^*, deletion of either one or the other *UAS* resulted in some sort of a *r.e.* phenotype (Fig. 3d). In these cases, we could not assign the suppressor effect unequivocally to one gene or direction. For the further verification of the effective genes, we used *RNAi* knockdown.

### RNAi knockdown of the effective genes alleviates apoptosis suppression

We tested whether a *UAS-RNAi* transgene, which specifically silences the *DEP*-bearing gene or one of the neighbor ones, can partly or entirely restore the *r.e.* phenotype in the *GMR-Gal4>UAS-RNAi, suppr^DEP^, UAS-p53* genotype. Therefore, we crossed flies carrying a *DEP* mutant and a *UAS-RNAi* construct to the *GMR-Gal4; UAS-p53* tester combination, and the results are summarized in Table 1. The *r.e.* phenotypes were scored according to the *r.e.* scale (Supplementary Fig. 2). Gal4-induced expression of the *RNAi* transgenes by themselves did not cause *r.e.* phenotype (not shown).

**Table 1.** Effect of RNAi silencing on the apoptosis suppression in the *GMR-Gal4>suppr^DEP^*, *UAS-RNAi*, *UAS-p53* combination.

*DEP* insertion mutant	Tested genes in the *DEP* insertion region	Effective *RNAi* constructs*^a^* (score > 1)	Ineffective *RNAi* constructs*^a^* (score < 1)
*Orct2^DEP54^*	*Orct2*		VDRC 106681 BDSC 57583
	*jar*		VDRC 37534 VDRC 37535 VDRC 108221 BDSC 28064
*Polr2M^DEP105^*	*PolR2M*	BDSC 42917 (1–2)	
	*CG5250*	BDSC 57432 (1–2)	
*Pka-R2^DEP327^*	*Pka-R2*	NIG-FLY 15862R2 (4) BDSC 27680 (3–4) BDSC 34983 (4)	
	*TER94*		BDSC 31968
	*CG12128*		BDSC 33997
	*CG1407*		BDSC 50601
*Rga^DEP375^*	*Rga*		BDSC 57549
	*asl*		BDSC 38220
	*Atu*	VDRC 106074 (2–3)	
	*Spec2*		BDSC 65206
*stg^DEP871^*	*stg*	BDSC 36094 (2)	
*crol^DEP1004^*	*cro*l		BDSC 44643
	*CG14937*		BDSC 31483
	*CycY*		BDSC 34009
	*esc*		BDSC 31618
*Fak^DEP2107^*	*Fakl*		VDRC 17957 BDSC 29323 BDSC 33617 BDSC 35357
	*CalpA*		BDSC 29455
	*Spt5*	NIG-FLY 7626R-3 (3)	
		BDSC 34837 (4)	

BDSC, Bloomington Drosophila Stock Center (Bloomington, Indiana); VDRC, Vienna Drosophila Resource Center (Vienna, Austria); NIG-FLY, National Institute of Genetics (Mishima, Japan).

^*a*^Only those *RNAi* constructs were tested, which were located on different chromosomes from the *DEP* insertions. Specification of the *RNAi* stocks is given with the stock center name and stock number. The numbers in brackets mean the score of the *r.e.* phenotype (see Supplementary Fig. 2).

In the case of *Pka-R2^DEP327^*, the results were straightforward: all 3 *Pka-R2* silencing *RNAi* transgenes tested restored the *r.e.* phenotype, verifying that the suppressor effect was really caused by the overexpression of *Pka-R2*. At the same time, silencing the neighbor genes *TER94*, *CG12128*, and *CG1407* had no effect (Table 1). In the case of *Fak^DEP2107^*, the *UAS* promoter deleting experiments suggested *Spt5* to be the gene responsible for the suppressor effect (Fig. 2). Accordingly, *RNAi* knockdown of *Spt5* brought back the *r.e.* phenotype in the *GMR-Gal4*>*Fak*^DEP2107^*, UAS-Spt5i, UAS-p53* combination (Table 1). In addition, an *Spt5* overexpressing transgene effectively suppressed the *r.e.* in the *GMR-Gal4*>*UAS-Spt5, UAS-p53* combination (not shown). All these results prove that the *Spt5* gene is an apoptosis suppressor.

In the case of *Rga^DEP375^*, the *UAS^loxP^* pointing in the direction of the *Atu* gene shows the suppressor activity (Fig. 2). In accordance with this, the *Atu*-silencing *RNAi* transgene restored the *r.e.* phenotype but silencing *Rga* and the neighboring genes *asl* and *Spec2* had no effect (Table 1).

In *stg^DEP871^*, the *DEP* element sits in the first exon near the 5′-end of *stg*, and the deletion test showed that, to some extent, both *UAS* promoters were responsible for the apoptosis suppression. The *UAS^FRT^* initiates transcription toward *CG45544*, an unknown gene nearby (Fig. 2). There was no *RNAi* construct available for this gene so we could not test the possible influence of *CG45544* on the suppressor effect. However, an *RNAi* transgene silencing *stg* moderately reduced the suppressor effect of *stg^DEP871^* (Table 1). We also tested a *UAS-stg* construct and detected that the overexpression of *stg* was able, albeit weakly, to suppress the *r.e.* phenotype in the *UAS-stg/GMR-Gal4; UAS-p53/+* combination (not shown). Taken together, one can suppose that in *stg^DEP871^*, the simultaneously induced expression of *stg* and *CG45544* could additively suppress apoptosis.

In the case of *Polr2M^DEP105^*, the *DEP*-bearing *Polr2M* and the neighbor gene *CG5250* were separately silenced. As it revealed, both tested *RNAi* transgenes weakened the suppression of apoptosis to some extent, but their effect was not strong (Table 1). This again suggests an additive suppressive effect of the 2 genes.

### RT-qPCR survey of gene activation by the *GMR-Gal4* driver

**Fig. 4.**
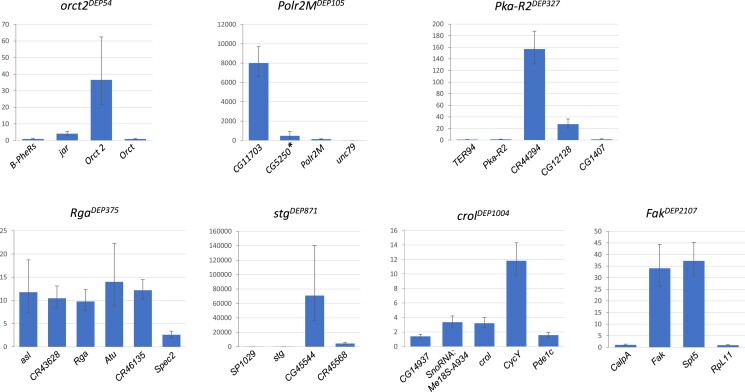
Gal4-induced activity of the genes bearing the *DEP* insertion and the genes in the neighborhood. The columns represent the fold change of the gene expression measured in *GMR-Gal4*>*Suppr^DEP^, UAS-p53* vs *GMR-Gal4>UAS-p53*. For the numerical results, see Supplementary Table 3, *uninduced control: *w; Polr2M^DEP105^/TM3*.

Supposing that the Gal4-induced overexpression of the gene bearing the *DEP* insertion can spread over the neighbor genes in the region, and their elevated expression may also contribute to the suppressor phenotype, in a RT-qPCR experiment, we systematically tested the expression levels of the nearby genes as well. The results of this survey are summarized in Fig. 4 and Supplementary Fig. 6 and Table 3.

In addition to each gene with the *DEP* insert, Supplementary Table 3 contains the genes in the surrounding region and shows the distances in kb between the genes' transcription start sites and the *DEP* insertion site, as well as the fold change values of their *GMR-Gal4*-induced expression levels. The expression levels were measured with *UAS-p53* in the background (*GMR-Gal4>suppr^DEP^, UAS-p53* vs *GMR-Gal4>UAS-p53*). For the statistical evaluation of the results, see Supplementary Table 3.

The *DEP* as a *P* element derivative mostly inserts itself into or near to the 5′-end of the genes. Consequently, 1 of the 2 *UAS* promoters can always start the downstream transcription of the gene resulting in a supposedly normal mRNA. Compared to the uninduced “basic activity,” *GMR-Gal4* can induce a significantly elevated expression of the *DEP*-bearing gene, and the activating effect can spread to the nearby genes as well. In general, the level of activation decreased with the growing distance from the *DEP* insert, but the actual values varied depending on the gene and the region (Fig. 4; Supplementary Fig. 6 and Table 3).

The fold degree of activation largely depended on the basic, uninduced level of the gene activity (see in FlyAtlas, www.flyatlas2.org): when it was very low in general, the Gal4-induced expression could reach high or extremely high relative levels. For example, for *stg^DEP87^*^1^, the *GMR-Gal4*-induced fold change was 146 times, while in the vicinity, *CG45544* (distance from *DEP* insertion site 1.6 kb) and *CR45568* (distance 21.8 kb) were induced by 7 × 10^4^ and 4 × 10^3^ times, respectively (Fig. 4; Supplementary Fig. 6 and Table 3).

### Apoptosis suppressor mutants in coexpressed gene clusters

We compared the chromosomal location of the genes in the *DEP* insertion neighborhoods with that of the known coexpressed gene clusters in the *Drosophila* genome (Spellman and Rubin 2002). Supplementary Table 4 shows that in 3 mutants (*Polr2M^DEP105^*, *Rga^DEP375^*, and *Fak^DEP2107^*), the *DEP-*bearing genes and their neighbors were included in 3 separate coexpressed clusters. In addition, 2 other mutants, *Orct2^DEP54^* and *crol^DEP1004^*, are located near the boundary of further 2 clusters. In *Polr2M^DEP105^*, the promoter deletion and the *RNAi* experiments together identified *Polr2M* and *CG5250* genes that were able to suppress the p53-induced apoptosis to some extent, when overexpressed. As it revealed, at least 2 genes of the coexpressed cluster hit by the *Polr2M^DEP105^* insertion could be involved in the process of apoptosis regulation. Whether the other genes in this and other clusters have similar ability or could influence the antiapoptotic activity of the *DEP* neighborhood genes remains to be seen.

## Discussion

In the present study, we identified genes by genomic insertions of the *DEP* element through their ability to suppress the *r.e.* phenotype induced by the *GMR-Gal4*-driven *p53*. We think that the main cause of the suppressor effect is the suppression of the cell death. However, the p53 as a transcription factor can directly or indirectly influence the expression of many genes, which may contribute to the given phenotype. For example, *p53* can induce p21 (Deiry *et al.* 1993; Fan *et al.* 2010) that arrests the cell cycle through different pathways (Engeland 2018, 2022) and also regulates other nonapoptotic cell death pathways like ferroptosis, entosis, and paraptosis (Bredesen *et al.* 2006; Liang *et al.* 2021; Yu *et al.* 2021). In addition, it was reported that overexpression of *p53* in the eye disturbed the differentiation of R7 photoreceptor neurons and cone cells that also resulted in *r.e.* phenotype. This suggests that the *r.e.* is caused by apoptosis and differentiation defects together (Fan *et al.* 2010). Both these processes can be suppressed by expression of p21*/dap* (Fan *et al.* 2010). If such processes are responsible for the *r.e*. phenotype, the suppressor genes we identified should inhibit some of them.

In the case of 4 *DEP* insertions, by in vivo selective elimination of one or the other *UAS* promoter of the *DEP* element, the gene from which the apoptosis suppression originates could be determined by the loss of its effect, while in the rest 3 cases, the gene responsible for the suppressor effect remained uncertain. It has to be noted that, even in the cases when the suppression of apoptosis could be assigned to 1 gene, the flies having a truncated *DEP* with the “suppressor *UAS*” only showed a weaker suppression of the *r.e.* phenotype than the original mutant bearing the intact *DEP* element (Fig. 3c). In these cases, the induction of transcription and the possible activation of the neighbor genes were obviously lopsided. This may hint at the possibility that the weaker suppressor effect would either be a result of the missing activity of the neighbor genes on the “silent” side of the truncated *DEP* insert or, if both *UAS* promoters are simultaneously activated in the intact *DEP* element, there is synergy between them, e.g. by mutually loosening up the chromatin structure, which would enhance the level of transcription of the “suppressor” gene.

In the *GMR-Gal4* (Glass Multimer Reporter) driver, the Gal4 is mainly expressed in the developing eye disk and the adult eye (Ollmann *et al.* 2000; Roman and Davis 2002; Yang *et al.* 2005). However, *GMR-Gal4* expression was detected in other tissues as well, namely in the brain, trachea, and leg disks (Li *et al.* 2012). In addition, Ray and Lakhotya (2015) found that the strong Gal4 expression (e.g. *GMR-Gal4* in homozygous) on its own can interfere with normal eye development resulting in some *r.e.* adult phenotype. To avoid these possible disturbing effects, we used only 1 copy of the *GMR-Gal4* driver in heterozygous condition that on its own did not interfere with the normal eye development in the genetic combinations used (Fig. 3a′).

However, if we have only 1 copy of *GMR-Gal4* in the combination, the number of the Gal4 binding sites can become critical. If too many *UAS* motifs compete for the limited amount of Gal4 protein, the Gal4-induced apoptosis and *r.e.* phenotype would become weaker, mimicking the suppression of apoptosis. In the heterozygous “tester” combination (*GMR-Gal4*>*DEP*, *UAS*-*p53*), there were 3 *UAS* motifs sharing the Gal4 and with all of the more than 2000 ineffective *DEP* insertions the animals showed the *r.e.* phenotype. If the combination contains 4 *UAS*s (e.g. *GMR-Gal4>suppr^DEP^, UAS-RNAi, UAS-p53*), the *r.e.* is still well visible (Table 1). Above this number, however, the *r.e.* phenotype begins to weaken. Hence, the genetic combinations, we used, contained only 4 *UAS*s at the maximum.

In the case of *stg^DEP871^* mutant, all the experiments, including promoter deletion (Fig. 3), *RNAi* silencing (Table 1), and overexpression of *stg*, pointed to the direction that *stg*, at least in part, is responsible for the suppression of apoptosis. This is in accordance with the fact that *stg* is the *Drosophila* ortholog of the *cdc25* phosphatase that is a key factor of mitosis progression and reported earlier to be able to inhibit apoptosis (Kylsten and Saint 1997; Fuhrmann *et al.* 2001; Cho *et al.* 2015). Interestingly, Ruiz-Losada *et al.* (2022) recently published their observation that seemingly opposes these above results. They found that overexpressing the *stg* gene in larval wing disks followed by X-ray irradiation acted in proapoptotic way. The exact relation of *stg* to apoptosis needs further investigation.

In mutant *Fak^DEP2107^*, both the promoter deletion and RNAi experiments suggested that the suppressor effect was exerted by *Spt5* instead of *Fak* (Figs. 2 and 3 and Table 1). This observation was not expected, since the mammalian ortholog gene FAK is a potent apoptosis suppressor (Sonoda *et al.* 2000; Kurenova *et al.* 2004).

Interestingly, neither of the genes we identified as apoptotic suppressor belongs to the IAP gene family. Only 3 IAP genes, *Diap1*, *Diap2* (Hay *et al.* 1995), and *Bruce* (Domingues and Ryoo 2012) were discovered in *Drosophila*, so the likelihood of a random hit by the *DEP* is very low, and we did not recover any insertion in them. Similarly, we did not find *DEP* mutants for other known apoptosis suppressor genes either: *Api5* (Morris *et al.* 2006); the MDM2 ortholog, *corp* (Chakraborty *et al.* 2015); and the BCL-2 prosurvival family member, *Buffy* (Colussi *et al.* 2000; Brachmann *et al.* 2000; Quinn *et al.* 2003). Genes inhibiting cell death are very important, and presently, intensive research is focused on them as potential targets of anticancer drugs like the Bcl-2 inhibitor venetoclax (Fairbrother *et al.* 2019). We hope that the genes we identified will also contribute to the progress of the field.

On the chromosomes of eukaryotic organisms, there are gene clusters in which the genes are coexpressed (Ben-Shahar *et al.* 2007; Michalak 2008; De and Babu 2010; Mihelčić *et al.* 2019). Some of the clusters contain genes with similar functions, while others have genes with diverse functions (Lercher *et al.* 2002). Such coexpressed gene clusters were found also in *Drosophila* (Boutanaev *et al.* 2002; Spellman and Rubin 2002; Stolc *et al.* 2004).

As it revealed, 3 out of the 7 mutants (*Polr2M^DEP105^*, *Rga^DEP375^*, and *Fak^DEP2107^*) and their neighbor genes are located in 3 separate coexpressed clusters, and 2 mutants (*Orct2^DEP54^* and *crol^DEP1004^*) are outside but very near to other separate clusters (Supplementary Table 4). This is particularly interesting in the case of *Polr2M^DEP105^* insertion, since we identified both *Polr2M* and *CG5250* in its neighborhood to be able to suppress the p53-induced apoptosis to some extent. It may suggest that at least one of the common goals of the cellular processes, in which the genes of this cluster are involved, could be the suppression of apoptosis, even without the activation of these genes by Gal4. We speculate that this suppression can be an additive effect of the activated genes around the *DEP* insertion. Whether the other genes in the cluster would exert similar effect, and the genes in the other clusters mentioned above could influence the suppression of apoptosis, needs further investigation.

The question promptly arises whether these genes near the *DEP* insertion site can really suppress apoptosis or they can influence the regulation of apoptosis in any respect. To this end, we conducted a survey in the literature for the genes tested in the RT-qPCR experiment. As the *Drosophila* genes are not so well characterized in this respect, we examined their human orthologs as well. The programmed cell death is one of the most important factors blocking the development of cancer; therefore, a lot of information and data can be found about the protein-coding human genes in this respect. Logically, if a gene has any antiapoptotic effect, its overexpression promotes cell proliferation and tumor development while its reduced activity has the opposite effect. Supplementary Table 5 shows the 7 *DEP*-bearing *Drosophila* genes and their close neighbors (26 genes) as well as their human orthologs (24 genes), see GeneCards the Human Gene Database (Stelzer *et al*. 2016). Altogether, according to the literature, 21 of these human genes possibly have antiapoptotic activity, 2 genes are proapoptotic, and 1 gene is uncertain in this respect. Our results in *Drosophila* call attention to the apoptosis suppressive effect of these genes.

Taken together, as our results suggest, in certain cases, not only the gene examined but other genes in the vicinity can also influence the regulation of the programmed cell death, especially if they are overexpressed ectopically. In general, while the effect of a single gene can be negligible, the combined effect together with the neighbor genes can add up to a significant level.

## Supplementary Material

jkae149_Supplementary_Data

## Data Availability

The strains and the *DEP* transposon are available upon request. All data confirming the conclusions of the article are included in the article, figures, and tables. Supplemental material available at G3 online.
