# Supplementary material for: Dominant suppressor genes of p53-induced apoptosis in Drosophila melanogaster
Source: G3 (Bethesda). 2024 Jul 10;14(9):jkae149. doi: 10.1093/g3journal/jkae149 (PMC11373661; doi:10.1093/g3journal/jkae149)
Supplement: jkae149_Supplementary_Data [file jkae149_supplementary_data.pdf]

# Supplementary Material

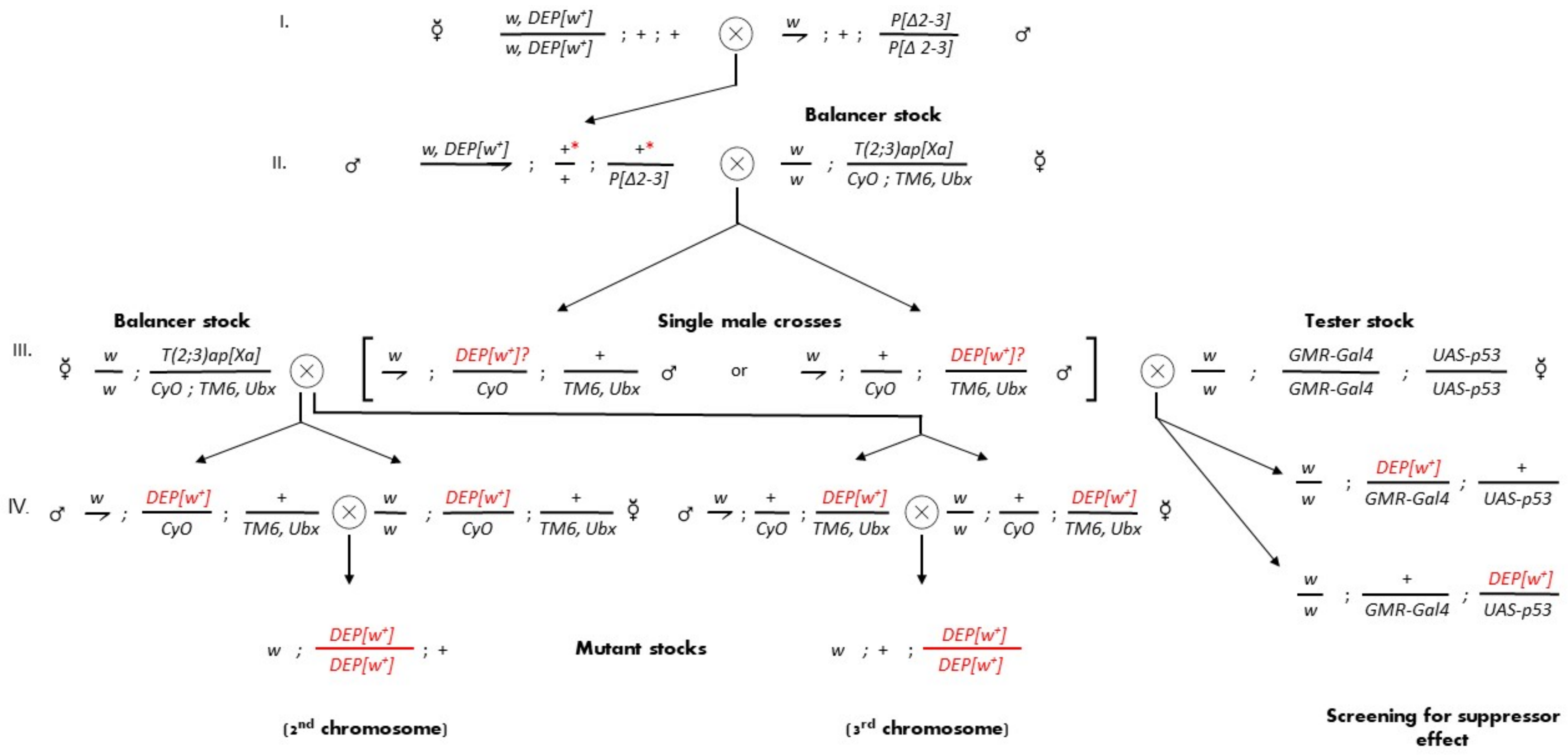


**Supplementary Fig. 1: Crossing scheme of *DEP* insertional mutant isolation.** Red stars mark the possible new DEP insertions in the male germline.

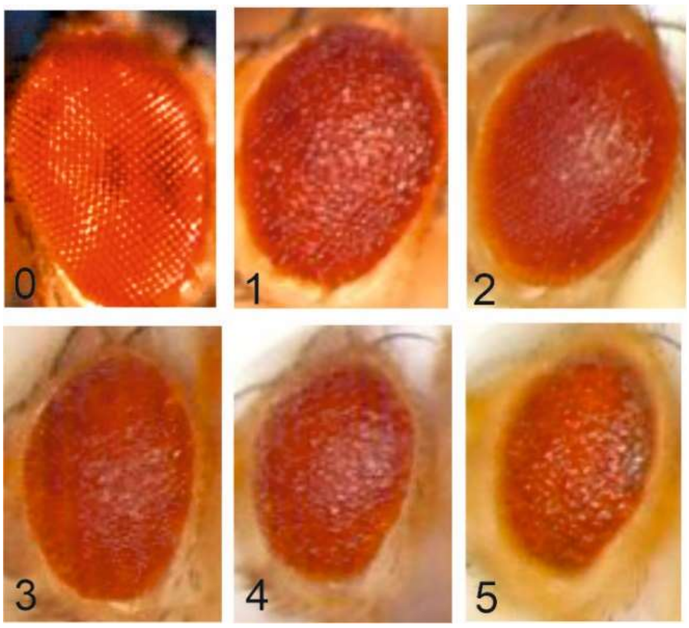


**Supplementary Fig. 2: Six grade rough-eye scale.** 0: wild type, 5: strongest rough-eye phenotype, 1-4: weaker grades of the rough-eye.

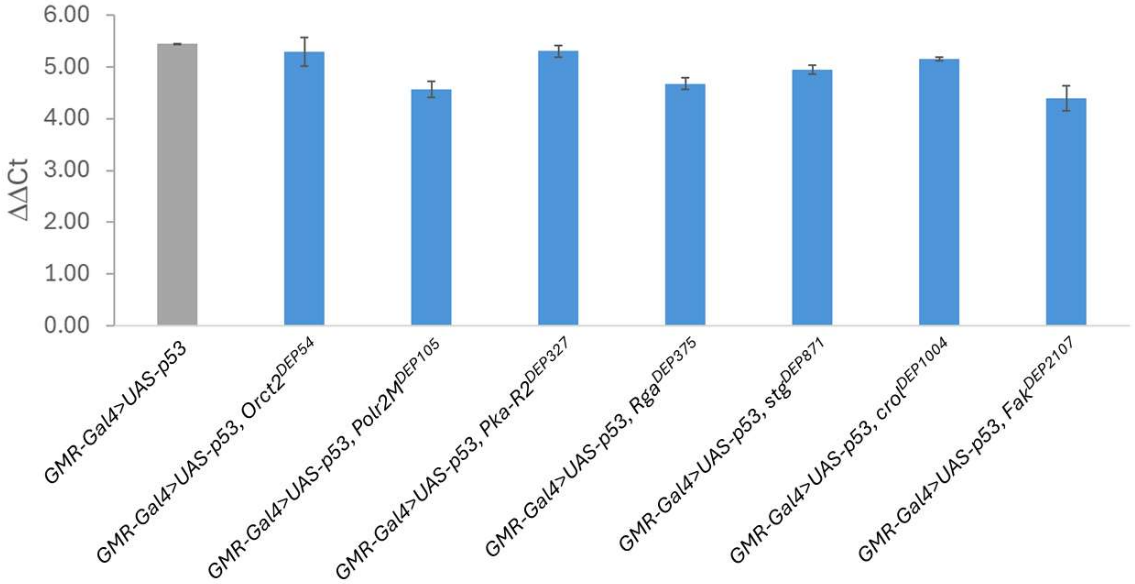


**Supplementary Fig. 3: Gal-4 induced level of p53 mRNA in the DEP-bearing mutants (*GMR-Gal4>Suppr$^{DEP}$*, *UAS-p53*; blue bars*)* compared to the original *p53* overexpressing *GMR-Gal4>UAS-p53* flies (grey bar).** Columns represent ΔΔCt values from RT-qPCR experiments performed on total RNA preparations from the heads of 3-day-old adults. For more details see the Materials & Methods section.

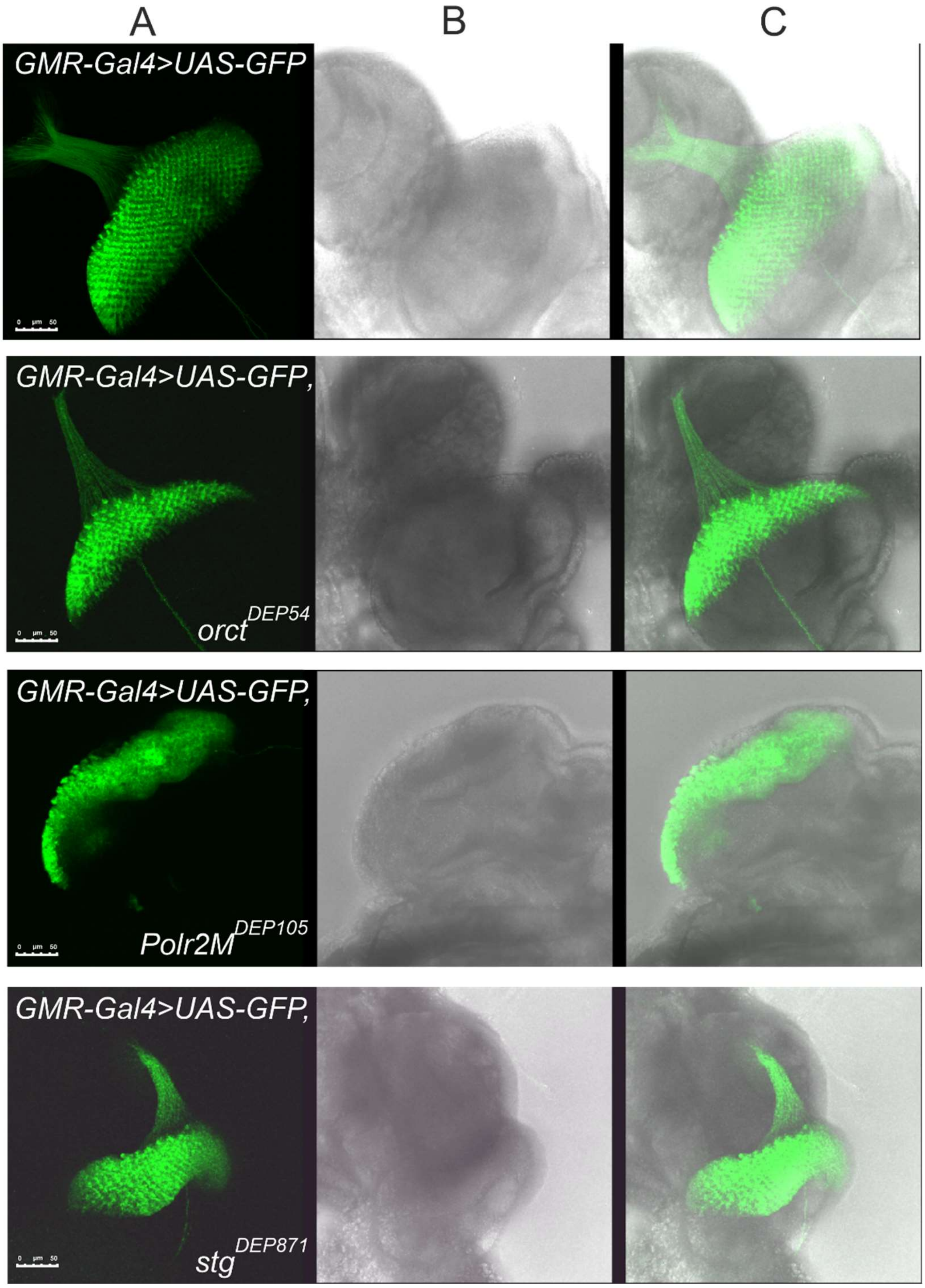


**Supplementary Fig. 4: Representative confocal images of *GMR-Gal4*-induced GFP signal in eye imaginal discs from 3$^{rd}$ instar larvae of the indicated genotypes.** GFP patterns demonstrate that the driving capacity of the *GMR-Gal4* driver is not changed on different *Suppr*$^{DEP}$ backgrounds (indicated in the lower right corner of the images) as compared to wild type background (*GMR-Gal4>UAS-GFP*). Column **A**: GFP signal, column **B**: transmission images, column **C**: merged. Images of native GFP signal were taken by HCX PL FLUOTAR 40x/0.75 objective. Scale bars represent 50 µm.

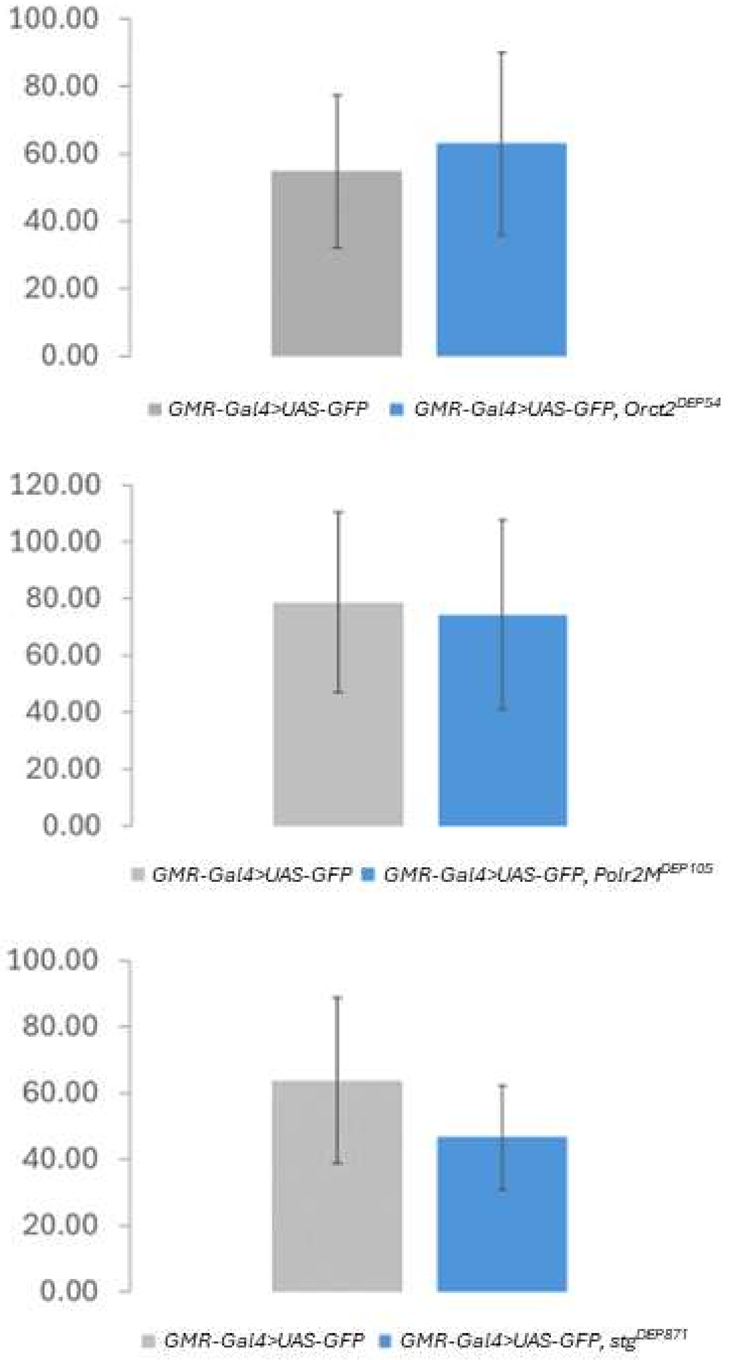


**Supplementary Fig. 5: Pairwise comparison of *GMR-Gal4*-induced GFP fluorescence signal intensity in 3rd instar larval eye discs from *Suppr*$^{DEP}$ mutants with their corresponding control ones.** For quantitative analysis the 3D volume was acquired by optical sections of the samples (3-9 eye discs/sample.) The images of compared eye discs of the *DEP*-bearing genotypes and their corresponding controls were captured from the same slide and at the same time. The fluorescence intensities of the Z-sections were averaged using Z-Projection in order to get all layers into consideration. The mean/std was calculated from the pixel values higher than 25 to exclude the background noise of the images.

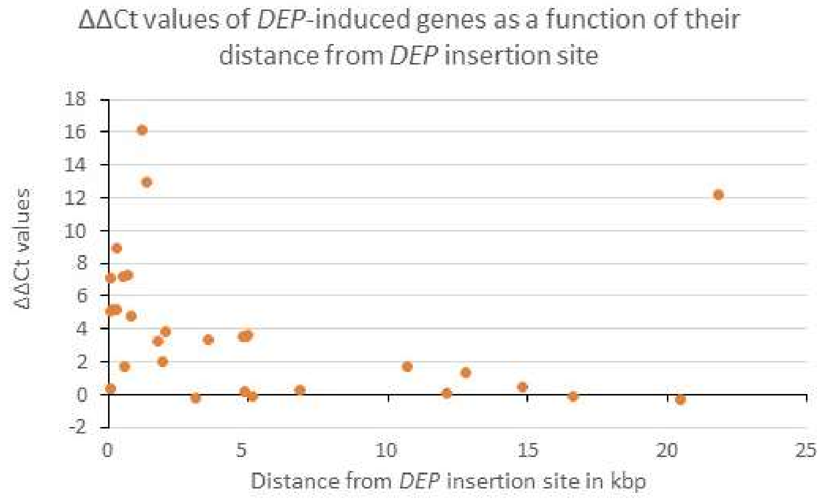


**Supplementary Fig. 6: ΔΔCt values from the RT-qPCR experiment representing the induction rate of genes as a function of their distance from the *DEP* insertion site.** The dots represent single genes.

| *DEP* insertion mutants | Distance of the *DEP* insertion from the transcription start site of the relevant gene | Absolute position on the chromosome arm | Flanking sequence of the insertion point[1] |
|---|---|---|---|
| ***orct2***$^{DEP54}$ | 136 | 3R: 24273164 | CAACATATTTGGGCAACACGTGTTGGCCGATAATTGCCGGAACCGAGA |
| ***Polr2M***$^{DEP105}$ | 69 | 3R: 19229531 | GATAATGTGATTCCTGCTCCGCTTAGCCGTATCCCGGGCACCAGCCAG |
| ***Pka-R2***$^{DEP327}$ | upstream 31 | 2R: 10026073 | AATTGCGGGTTGCTTAAATAGACTTGGTGCTCCCGCAAAAGCTCGCTC |
| ***Rga***$^{DEP375}$ | 1778 | 3R: 5611335 | ACGAGAAGCATGGGCAGAAAGGCTGTGGCAATTATTGCCGTTCCTCTT |
| ***stg***$^{DEP871}$ | 512 | 3R: 29255289 | CCGAAAGGCTGAACAAGAAGAGCTGTTGACTCGACGACGAACTGAGAA |
| ***crol***$^{DEP1004}$ | 556 | 2L: 11808883 | ACTACGAGAGACACCGCAAGATACAGATACAATGCGTGCTCGAGCCCG |
| ***Fak***$^{DEP2107}$ | 55 | 2R: 19437205 | AGTAATGGTTATTTTTAAAAATATGATTACTTATTTCTCCGTAGTAGC |

**Supplementary Table 1**: **Genomic positions of *DEP* insertions.** [1] Nucleotides in red indicate the 8-basepair duplications generated by the integration of the *DEP* element. The magnified nucleotides refer to the absolute position of the *DEP* insertion site in the DNA sequence of the relevant chromosome arm.

| Gene | Forward Primer | Reverse Primer |
|---|---|---|
| *asl* | gcccttaacataaattctacactgg | ccataatccgtccaagctct |
| *Atu* | acgccgcaaggaggtagt | ctacgcggagacccagag |
| *beta-PheRS* | cacgggtctgctggtattc | tcgaagggtcaatcttcagc |
| *CalpA* | ctttatcaacacccgcgaag | aaagttgaaggcacaatcagg |
| *CG11703* | cctaatcgtgtgcattgtgg | gcttattcggatcaatgatgg |
| *CG12128* | ggcaatagaaccaggcgtcagag | ccccgctgctttctacagctc |
| *CG1407* | ccgcggtaatagcttggtc | atgcggttctccgagttg |
| *CG14506* | tttttatgccatttacgaagaaatc | ccaaaaaggaagacaagtagtgtg |
| *CG14937* | gacgtccgcaagcgatac | caaagccaggacatcgtca |
| *CG3773* | gacctgaagacccttacctatcc | cagacgctttcccttgtcc |
| *CG45544* | cgcacagtgtggcaaaatac | acgcttttgattccgcttc |
| *CG5250* | gcgtgggctaagaacatacc | atgaactggtgccgtcaga |
| *CR43628* | tgttcaccatggtcgctggtatg | cggtgtacatcacaaaagcccaac |
| *CR44294* | agtttgacatctccgccgttagac | aggtcgatccgactgtccactg |
| *CR45568* | cggggatctcgaaataagtg | gctcctagctgtttgtcagca |
| *CR46135* | ggagtcgcgcagtgccttg | acgagggctccgacttcgag |
| *crol* | agccacaacatcagcacaac | gtcgactgctttcgctgag |
| *CycY* | gtgcgatcatcacgctagtc | ttgaggcgaggagaatgg |
| *Fak* | aacgctggattgtgaatatcg | caggtttttggcgtccatag |
| *FoxK* | cgcccagtcacatggataa | ggtgctgattgcaacacaat |
| *fzr* | cgcagaacgtactggctgt | agatcacacaggcgggtaac |
| *jar* | ccccaggatcacgatgac | aacgcgttttcaaattgtcc |
| *Me18S-A934* | catttacctcttgagcctaatttgcat | agttttggggggcttgctctcac |
| *Orct* | gaggcctttgtgatcatcg | gctgctcgtagatttcattgg |
| *p53* | acatgagccggtctgtaacc | gcttggggcacgtacatatt |
| *Orct2* | tcgatgagggagaaaactcg | tcatccagctcctgatcctc |
| *Pde1c* | agcaggccgtcaaagatg | tttccggcttgctttcct |
| *Pka-R2* | aactcacaggatgccgatg | ctcggcgaaaactgatttg |
| *Rga* | tagctggccggacaacag | cgttcctggataggagttgg |
| *RpL11* | tctggacttctacgtcgtcctc | cttcatggcatcctccttg |
| *SP1029* | caagcaggactcgatgtgg | gtccttggtgagcggatg |
| *Spec2* | cggagcaacaagagatttcc | gttgtgtagcgtgggctatg |
| *Spt5* | cccagccctggatacaatc | cagagtgccaggagtttgc |
| *stg* | cagcatggattgcaatatcagta | acgacagctcctcctggtc |
| *TER94* | aaaatcagaactccgcgatg | cgctagtaatctccggcact |
| *unc79* | tccctggctaaacaatgctc | cgcattctgtgaactccttg |

**Supplementary Table 2: Primers used in RT-qPCR experiments.**

| *orct2* DEP54 | *β-PheRs* | *jar* | *orct2* | *orct* |
|---|---|---|---|---|
| distance in kbp | 20.5 | 1.9 | 0.136 | 3.1 |
| fold change | 0.8 | 4.1 | 37 | 0.8 |
| ΔΔCt | -0.26 | 2.03 | 5.19 | -0.24 |
| S.D. of ΔΔCt-s | 0.61 | 0.43 | 0.77 | 0.57 |
| Significance **P** value | 0.5520 | 0.0023 | 0.0022 | 0.5599 |

| *Polr2M* DEP105 | *CG11703* | *CG5250** | *PolR2M* | *unc79* |
|---|---|---|---|---|
| distance in kbp | 1.4 | 0.255 | 0.069 | 16.6 |
| fold change | 8000 | 476 | 135 | 0.9 |
| ΔΔCt | 12.97 | 8.90 | 7.08 | -0.08 |
| S.D. of ΔΔCt-s | 0.27 | 0.97 | 0.30 | 0.26 |
| Significance **P** value | 0.00001 | 0.0031 | 0.0006 | 0.6726 |

| *Pka-R2* DEP327 | *TER94* | *Pka-R2* | *CR44294* | *CG12128* | *CG1407* |
|---|---|---|---|---|---|
| distance in kbp | 36.9 | 0.031 | 0.685 | 0.766 | 6.8 |
| fold change | 0.9 | 1.3 | 157 | 28 | 1.2 |
| ΔΔCt | -0.18 | 0.39 | 7.30 | 4.78 | 0.31 |
| S.D. of ΔΔCt-s | 0.29 | 0.28 | 0.26 | 0.40 | 0.86 |
| Significance **P** value | 0.4173 | 0.1044 | 0.000002 | 0.0014 | 0.6224 |

| *Rga* DEP375 | *asl* | *CR43628* | *Rga* | *Atu* | *CR46135* | *Spec2* |
|---|---|---|---|---|---|---|
| distance in kbp | 4.8 | 3.5 | 1.8 | 2.0 | 5.0 | 12.8 |
| fold change | 12 | 10 | 10 | 14 | 12 | 2.6 |
| ΔΔCt | 3.56 | 3.39 | 3.29 | 3.81 | 3.61 | 1.38 |
| S.D. of ΔΔCt-s | 0.67 | 0.32 | 0.33 | 0.67 | 0.25 | 0.38 |
| Significance **P** value | 0.0147 | 0.0001 | 0.0001 | 0.0131 | 0.0001 | 0.0203 |

| *stg* DEP871 | *SP1029* | *stg* | *CG45544* | *CR45568* |
|---|---|---|---|---|
| distance in kbp | 4.8 | 0.512 | 1.2 | 21.8 |
| fold change | 1.1 | 146 | 70933 | 4522 |
| ΔΔCt | 0.13 | 7.19 | 16.11 | 12.14 |
| S.D. of ΔΔCt-s | 0.24 | 0.64 | 0.99 | 0.31 |
| Significance **P** value | 0.4919 | 0.0008 | 0.00002 | 0.00002 |

| *crol* DEP1004 | *CG14937* | *Me18S-A934* | *crol* | *CycY* | *Pde1c* |
|---|---|---|---|---|---|
| distance in kbp | 14.8 | 10.7 | 0.556 | 4.9 | 119.7 |
| fold change | 1.4 | 3.4 | 3.2 | 12 | 1.6 |
| ΔΔCt | 0.48 | 1.75 | 1.68 | 3.56 | 0.64 |
| S.D. of ΔΔCt-s | 0.26 | 0.32 | 0.31 | 0.27 | 0.30 |
| Significance **P** value | 0.0752 | 0.0035 | 0.0014 | 0.0003 | 0.0341 |

| *Fak* DEP2107 | *CalpA* | *Fak* | *Spt5* | *RpL11* |
|---|---|---|---|---|
| distance in kbp | 12.1 | 0.055 | 0.278 | 5.1 |
| fold change | 1.1 | 34 | 37 | 0.9 |
| ΔΔCt | 0.10 | 5.09 | 5.22 | -0.16 |
| S.D. of ΔΔCt-s | 0.35 | 0.38 | 0.28 | 0.37 |
| Significance **P** value | 0.6868 | 0.0015 | 0.0001 | 0.5512 |

**Supplementary Table 3: Distance (from the relevant *DEP* insertion site) and induction rate (calculated from the RT-qPCR results) of the tested genes together with statistical evaluation.** Distance in kbp means the distance between the genes' transcription start site and the *DEP* insertion site. Fold change values were calculated as a ratio between expression levels of *GMR-Gal4>Suppr*$^{DEP}$*, UAS-p53* and the *GMR-Gal4>UAS-p53* control genotype, using the formula (fold change=$2^{\Delta\Delta Ct}$, see Materials and Methods). * Uninduced control genotype: *w; Polr2M*$^{DEP105}$*/TM3.* S.D. stands for standard deviation. For P value calculation normalized Ct (ΔCt) values were used in students' t-test (two-tailed, unequal variance), n=3.

| The gene bearing the *DEP* insert | Genes in the neighborhood | The genes' functions | The genes' chromosomal localization | Co-expressed neighborhood regions (Spellman and Rubin, 2002) |
|---|---|---|---|---|
| *Orct2*$^{DEP54}$ | *jar* | MyosinVI, intracellular transporting molecular motor, binding actin filaments and microtubules | 3R: 24,252,826 – 24,271,233 | **3R: 24,276,260 – 24,333,316** |
| | *Orct2* | Organic cation transporter | 3R: 24,273,029 – 24,275,728 | |
| | ***Orct*** | Organic cation transporter | **3R: 24,276,260 – 24,278,792** | |
| *Polr2M*$^{DEP}$ | ***CG11703*** | Part of intracellular sodium:potassium-exchanging ATPase complex | **3R:19,226,903 – 19,228,180** | **3R: 19,164,211 – 19,248,136** |
| | ***CG5250*** | Part of the intracellular sodium:potassium-exchanging ATPase complex | **3R:19,228,217 – 19,229,276** | |
| | ***PolR2M*** | RNA polymerase II subunit M | **3R:19,229,463 – 19,230,832** | |
| | ***unc79*** | Protein involved in circadian locomotor rhythms | **3R:19,229,463 – 19,230,832** | |
| *Rga*$^{DEP375}$ | ***asl*** | Component of the centriole | **3R:5,603,236 – 5,606,543** | **3R: 5,588,873 – 5,624,125** |
| | ***CR43628*** | Antisense lncRNA of Rga | **3R:5,607,799 – 5,609,810** | |
| | ***Rga*** | mRNA deadenylase, part of the CCR4-NOT complex | **3R:5,607,275 – 5,613,112** | |
| | ***Atu*** | Involved in transcription elongation, part of Cdc73/Paf1 complex | **3R:5,613,358 – 5,615,939** | |
| | ***CR46135*** | Antisense lncRNA, unknown | **3R:5,613,280 – 5,616,308** | |
| | ***Spec2*** | CDC42 small effector 2, inducing actin filament assembly | **3R:5,616,644 – 5,624,125** | |
| *crol*$^{DEP1004}$ | *CG14937* | Uncharacterized protein | 2L:11,792,412 – 11,794,086 | **2L: 11,496,804 – 11,792,365** |
| | *crol* | Zinc-finger transcription factor regulating *wg* transcription and cell cycle progression in *Drosophila* . | 2L:11,794,327 – 11,809,438 | |
| | *CycY* | Positive regulator of cyclin-dependent protein serine/threonine kinase activity | 2L:11,810,092 – 11,813,780 | |
| | *Pde1c* | 3',5'-cyclic nucleotide phosphodiesterase with a dual specificity for the second messengers cAMP and cGMP | 2L: 11,814,156 – 11,928,572 | |
| *Fak*$^{DEP210}$ | ***CalpA*** | Calcium-dependent modulatory protease | **2R:19,425,113 – 19,430,564** | **2R: 19,425,113 – 19,500,000** |
| | ***Fak*** | Focal adhesion kinase, a Non-Receptor Tyrosine kinase | **2R:19,430,659 – 19,437,259** | |
| | ***Spt5*** | Part of the DSIF complex regulating transcription elongation | **2R: 19,437,483 – 19,441,898** | |
| | ***RpL11*** | Component of large ribosomal subunit | **2R: 19,442,342 – 19,443,825** | |

**Supplementary Table 4: Distribution of the *DEP*-bearing genes and their neighborhoods relative to the positions of the co-expressed gene clusters on the *Drosophila* chromosomes** (Spellman and Rubin 2002)**.** Genes highlighted in dark grey and light grey are within or very near to a co-expressed region, respectively. *Pka-R2*$^{DEP327}$ and *stg*$^{DEP871}$ and their neighborhoods are far from any gene cluster, and therefore not included in the table.

| ***Drosophila* genes** | | **Human orthologs** | | | | |
|---|---|---|---|---|---|---|
| **The gene bearing the *DEP* insert** | **Genes in the neighborhood** | **Human genes** | **Gene function** | **Relation to apoptosis or cancer** | **References** (Pubmed ID) | **Remarks** |
| ***Orct2***$^{DEP54}$ | *β-PheRs* | FARSB | Phenylalanyl-tRNA synthetase beta | Anti-apoptotic ? | **Lu et al. 2014** (PMID: 25427601) | In *Drosophila* , Beta-Phe-RS mutations reduce cell proliferation and promote cell death. |
| | *jar* | Myo6 | Non-muscle myosin | Anti-apoptotic ? | **Wang et al. 2016** (PMID: 27515005) and **You et al. 2016** (PMID: 27044563) | Expression of MYO6 was higher in gastric cancer tissues than in the normal tissues. Knockdown of MYO6 increased the expression of apoptosis-related proteins Bax and cleaved Caspase-3, decreased Bcl-2 expression, and induced apoptosis. |
| | *Orct2* | SLC22A5 | SLC22A5 is an integral membrane protein which functions both as an organic cation transporter and as a sodium-dependent high affinity carnitine transporter. The encoded protein is involved in the active cellular uptake of carnitine, an important antioxidant. | Anti-apoptotic ? | **Vescovo et al. 2002** (PMID: 12176737) | L-carnitine can prevent apoptosis of skeletal muscles cells. |
| | | | | | **Qi et al. 2006** (PMID: 16328043) | In human lymphoma cells, L-carnitine is a potent anti-apoptotic agent inhibiting caspase-3 activity. |
| | *Orct* | SLC22A4 | Organic cation transporter, transmembrane ergothionein transport | Anti-apoptotic ? | **Fu and Shen 2022** (PMID: 35370675) | SLC22A4 is a specific transporter of ergothionein, an important antioxidant. |
| | | | | | **Jang et al. 2004** (PMID: 15036348) | Ergothionein, a diet-derived antioxidant, attenuates apoptosis caused by Abeta, preferentially by eliminating peroxynitrite derived from the neurotoxic peptide. |
| ***Polr2M***$^{DEP105}$ | *CG11703* | ATP1B1 | ATPase Na+/K+ transporter subunit β1. Maintenance of inorganic cation homeostasis through transmembrane K+ import/Na+export. | Anti-apoptotic ? | **Liu et al. 2016** (PMID: 27507851) and **Xu et al. 2010** (PMID: 20460749) | ATP1B1 expression is elevated in hepatocellular carcinoma cells. Knockdown inhibits cell proliferation and promotes oumabain- or cardiac steroid-induced apoptosis. |
| | *CG5250* | | | | | |
| | *PolR2M* | POLR2M (Protein: RINL1A) | RNA polymerase II subunit M. Negative regulator of transcription initiation by the Mediator complex. | Anti-apoptotic ? | **Jishage et al. 2020** (PMID: 32381628) | Knockout of POLR2M in hepatocytes causes injury-like reactions, activation of p53 signaling pathway, cell death and cell cycle re-entry. |
| ***Pka-R2***$^{DEP327}$ | *TER94* | VCP | VCP (Valosin Containing Protein) is part of the ubiquitin-proteasome system. | Anti-apoptotic ? | **Braun and Zischka 2008** (PMID: 18284922) | Mutations and depletion of Cdc48/VCP cause apoptosis whereas increased levels of this protein provide an anti-apoptotic effect. |
| | *Pka-R2* | PRKAR2A | Protein Kinase cAMP-Dependent Type II Regulatory Subunit Alpha | Pro- or anti-apoptotic ? | **Insel et al. 2012** (PMID: 21385327) and **Saloustros et al. 2015** (PMID: 26608815) | Protein kinase A can induce or inhibit cell proliferation, tumor progression or apoptosis depending on the regulatory subunits and/or interaction with other genes. |
| | *CG12128* | SPOUT1 | SPOUT domain-containing methyl-transferase 1 | Not determined | | |
| | *CG1407* | ZDHHC2 | Zn-finger DHHC-palmitoyl-S-tranferase | Pro-apoptotic ? | **Ko and Dixon 2018** (PMID: 30232163) and **Yan et al. 2013** (PMID: 23457560 | ZDHHC2 deleted in hepatocellular carcinoma; high expression of ZDHHC2 is a favorable prognostic marker in gastric cancer and renal cancer. |
| ***Rga***$^{DEP375}$ | *asl* | CEP152 | CEP152 is a core protein of the centrosome, a major microtubule-organizing center of animal cells that influences cell shape, polarity, and motility, and has a crucial function in cell division. | Anti-apoptotic ? | **Kalay et al. 2010** (PMID: 21131973) | Seckel cells homozygous for a mutation in CEP152 showed an overall increased sensitivity to oxidative stress and responded to this stress with increased apoptosis. |
| | | | | | **Bazzi and Anderson 2014** (PMID: 24706806) | CEP152 (centrosomal protein-152) is a gene required for centriole biogenesis. Embryos homozygous for a mutation in CEP152 showed increased apoptosis and p53 expression. |
| | | LAMA2 | LAMA2 is a subunit of Laminin. Laminins are glycoproteins with both common and specific functions. One common and most important function is that they interact with receptors anchored in the plasma membrane of cells thereby regulating multiple cellular activities and signaling pathways. | Anti-apoptotic ? | **Martins et al. 2024** (bioRxiv preprint, doi.org/10.1101/2024.01.20.576409) | LAMA2 codes for a subunit of laminin. Increased apoptosis is a major pathogenic mechanism in congenital muscular dystrophy caused by LAMA2 deficiency. Even though apoptosis is a hallmark of LAMA2-congenital muscular dystrophy, and its inhibition improved the outcome of the disease, it is a consequence of the disease rather than a primary cause. |
| | *Rga* | CNOT2 | mRNA decay is initiated by shortening of the polyA tail. The NOT complex is a major cytoplasmic deadenylase. | Anti-apoptotic ? | **Kentaro et al. 2011** (PMID: 21299754) | Suppression of CNOT2 expression markedly decreased the survival rate and increased apoptosis. |
| | | | | | **Kim et al. 2020** (PMID: 31894259) | CNOT2overexpression in TRAIL sensitive H460 cells enhanced the survival rate and decreased apoptosis. |
| | *Atu* | LEO1 | LEO1 is a component of the PAF complex (PAF1C) PAF1C associates with RNA polymerase II and is involved in transcriptional elongation. | Anti-apoptotic ? | **Chong et al. 2014** (PMID: 24686170) | LEO1 is an important mediator of PRL-3 oncogenic activities in acute myeloid leukemia (AML). Inhibition of Leo1 reverses PRL-3 oncogenic phenotypes in AML, impedes cell proliferation and induces significant apoptosis. |
| | | | | | **Gerlach et al. 2017** (PMID: 29078288) | In *Drosophila* , LEO1 helps recruit *Drosophila* Myc to promoters. |
| | *Spec2* | CDC42SE2 | CDC42SE2 is involved in the organization of the actin cytoskeleton by acting downstream of CDC42, a small GTPase of the Rho family, inducing actin filament assembly. | Anti-apoptotic ? | **Stengel and Zheng 2011** (PMID: 21515363) | Cdc42 is required for both G1-S phase progression and mitosis. Cdc42 is overexpressed in several human cancers. |
| | | | | | **Schoenherr et al. 2012** (PMID: 22615583) | Activating mutations in Cdc42 are transforming, while inhibition of Cdc42 activity can impinge on cellular transformation, and promotes apoptosis. |
| ***stg***$^{DEP871}$ | *SP1029* | ANPEP | Zinc metallo-amino-peptidase | Anti-apoptotic ? | **Azimi et al. 2017** (PMID: 29048432) | Targeting ANPEP Induced apoptosis. |
| | *stg* | CDC25A,B,C | CDC25s are dual specificity phosphatases acting upon tyrosine or serine/threonine residues. | Anti-apoptotic | **Cho et al. 2015** (PMID: 25633196) | Cdc25s are potential oncogenes overexpressed in many human cancers. Cdc25C suppresses apoptosis signal-regulating kinase1 (ASK1) and inhibits ASK1-mediated apoptosis. |
| | *CG45544* | unknown | uncharacterized | | | |
| ***crol***$^{DEP1004}$ | *CG14937* | unknown | Substrate adapter for SCF E3 ubiquitin ligase complexes. | | | |
| | *crol* | ZNF569 | Zinc-finger transcription factor regulating wg transcription and cell cycle progression in *Drosophila* . | Pro- or anti-apoptotic ? | **Huang et al. 2006** (PMID: 16793018) | ZNF569 protein acts as a transcriptional repressor and suppresses MAPK signaling pathway. |
| | | | | | **Wada and Penninger 2004** (PMID: 15077147) and **Yue and López 2020** (PMID: 32231094) | The regulation of apoptosis by MAPKs is complex and often controversial. |
| | | | | | **Mitchell et al. 2008** (PMID: 18614577) | In Drosophila, crol is required for cell cycle progression: crol mutant clones have reduced cell cycles and are removed by apoptosis, while upregulation of Crol overrides the Wg-mediated developmental cell cycle arrest. Crol acts to repress wg transcription. crol is anti-apoptotic. |
| | | | | | **Liu et al. 2019** (PMID: 31258729) | ZNF569 was hypermethylated in HNSCC (head and neck squamous cell carcinoma) tissues. In addition, the expression levels of ZNF569 mRNA and protein were significantly lower in HNSCC tissues and cell lines compared to their respective controls. Moreover, overexpression of ZNF569 inhibited the proliferation, migration and invasion of HNSCC cells. In this case, ZNF569 appears to be pro-apoptotic. |
| | *CycY* | CCNY | Positive regulation of cyclin-dependent protein serine/threonine kinase (CDK) activity. | Anti-apoptotic | **Shi et al. 2018** (PMID: 29557391) | Hepatocellular carcinoma (HCC) cells showed significantly increased expression of CCNY, cell proliferation, and a reduced rate of apoptosis. |
| | | | | | **Xie et al. 2018** (PMID: 29344296) | CDK16 is a cycline-dependent kinase which is activated by binding to cyclin Y (CCNY). CDK16 phosphorylates p53 at Ser315 and promotes the ubiquitination and subsequent degradation of p53. CDK16 accelerates cell proliferation and impairs apoptosis. |
| | *esc* | EED (Embryonic Ectoderm Development) | EED, EZH2 and SUZ12 are essential core components of the Polycomb Repressive Complex 2 (PRC2), a histone methyltransferase. | Pro- or anti-apoptotic ? | **Matsui et al. 2023** (PMID: 37553330) | Snip1 and PRC2 coordinate the intrinsic apoptosis program in the developing brain. Depletion of the PRC2 subunit EED reduces apoptosis and brain dysplasia in Snip1-depleted brain in vivo. |
| | | | | | **Ariës et al. 2018** (PMID: 30404791) | In T cell acute lymphoblastic leukemia, loss-of-function mutations of PRC2 core components (EZH2, EED, or SUZ12) were associated with mitochondrial apoptosis resistance. |
| | *Mal-B1* | SLC3A1 | Involved in the high-affinity, sodium-independent transport of cystine as well as neutral and dibasic amino acids. | Anti-apoptotic ? | **Jiang et al. 2017** (PMID: 28382174) | Elevated SLC3A1 expression accelerated the cysteine uptake and the accumulation of glutathione (GSH), leading to reduced reactive oxygen species (ROS). Decreased ROS reduced activity of the PP2Ac phosphatase, resulting in the activation of the AKT/PKB, protein kinase. Activated Akt mediates downstream responses, including cell survival (blocking apoptosis). |
| | *Mal-B2* | | | | | |
| | *Pde1c* | PDE1A | Member of the phospho-diesterase (PDE) family which catalyzes the hydrolysis of cAMP and cGMP. | Anti-apoptotic | **Abusnina et al. 2011** (PMID: 22045655) and **Ogawa et al. 2002** (PMID: 11964308) | PDE inhibitors cause the accumulation of cAMP, induction of p53 and p21 (WAF1/CIP1), G1 and G2/M cell cycle arrest, and increased apoptosis. |
| | | | | | **Nagel et al. 2006** (PMID: 16514069) | Reducing PDE1A function significantly attenuated vascular smooth muscle cell (VSMC) growth by decreasing proliferation via G1 arrest and inducing p53 activation and apoptosis. |
| ***Fak***$^{DEP2107}$ | *CalpA* | CAPN9 (calpain 9) | Calcium-dependent cysteine-type endopeptidase | Anti-apoptotic ? | **Momeni 2011** (PMID: 23507938) | Calpain promotes accelerated cell-cycle progression and anchorage-independent growth. |
| | | | | | **Łopatniuk and Witkowski 2011** (PMID: 21887410) | An increase in intracellular $Ca^{2+}$ level activates calpain. Activated calpains break down the cellular architecture leading to apoptosis. |
| | *Fak* | PTK2/FAK | FAK (Focal adhesion kinase) is a non-receptor protein tyrosine kinase. | Anti-apoptotic | **Kurenova et al. 2004** (PMID: 16514069) and **Kurenova et al. 2004** (PMID: 15121855) | FAK is over-expressed in a variety of human tumors. FAK can promote p53 degradation via enhanced Mdm2-dependent p53 ubiquitination. Attenuation of FAK expression in tumor cells results in apoptosis. |
| | | | | | **Golubovskaya and Cance 2011** (PMID: 21355845) | FAK and p53 are in a loop regulation: p53 protein inhibits FAK promoter activity and FAK protein also inhibits p53 transcriptional activity. |
| | *Spt5* | SUPT5H | Component of the DSIF complex. DSIF enhances transcriptional pausing to facilitate the assembly of an elongation competent RNA polymerase II complex. DSIF may also promote transcriptional elongation. | Anti-apoptotic | **Komori et al. 2009** (PMID: 19210550) and **Hartzog and Fu 2012** (PMID: 22982195) | Spt5 associates with Spt4. Spt4-Spt5 complex appears to couple chromatin modification states and RNA processing to transcription elongation. Spt5 is essential for cell growth, and Spt5 knockdown causes senescence and apoptosis. In Spt5 knockdown cells, the p53 signaling pathway is activated. |

**Supplementary Table 5: *Drosophila* genes, human orthologs and their possible relationship to apoptosis.** Provided that the functions of the *Drosophila* genes and their human orthologs are closely correlated, this table summarizes the genes' possible relationship to apoptosis. The numbers in brackets are the PubMed identification numbers in the cited references.
